# Human genes influence the interaction between *Streptococcus mutans* and host caries susceptibility: a genome-wide association study in children with primary dentition

**DOI:** 10.1038/s41368-019-0051-4

**Published:** 2019-05-30

**Authors:** Ying Meng, Tongtong Wu, Ronald Billings, Dorota T. Kopycka-Kedzierawski, Jin Xiao

**Affiliations:** 1 School of Nursing, University of Rochester, Rochester, NY USA; 2 Department of Biostatistics and Computational Biology, University of Rochester, Rochester, NY USA; 3 Eastman Institute for Oral Health, University of Rochester Medical Center, Rochester, NY USA

**Keywords:** Genome informatics, Microbiology

## Abstract

*Streptococcus mutans* is a well-known cause of dental caries, due to its acidogenicity, aciduricity, and ability to synthesize exopolysaccharides in dental plaques. Intriguingly, not all children who carry *S. mutans* manifest caries, even with similar characteristics in oral hygiene, diet, and other environmental factors. This phenomenon suggests that host susceptibility potentially plays a role in the development of dental caries; however, the association between host genetics, *S. mutans*, and dental caries remains unclear. Therefore, this study examined the influence of host gene-by-*S. mutans* interaction on dental caries. Genome-wide association analyses were conducted in 709 US children (<13 years old), using the dbGap database acquired from the center for oral health research in appalachia (COHRA) and the Iowa Head Start programmes (GEIRS). A generalized estimating equation was used to examine the gene-by-*S. mutans* interaction effects on the outcomes (decayed and missing/filled primary teeth due to caries). Sequentially, the COHRA and GEIRS data were used to identify potential interactions and replicate the findings. Three loci at the genes *interleukin 32* (*IL32), galactokinase 2 (GALK2)*, and *CUGBP, Elav-like family member 4* (*CELF4)* were linked to *S. mutans* carriage, and there was a severity of caries at a suggestive significance level among COHRA children (*P* < 9 × 10^−5^), and at a nominal significance level among GEIRS children (*P* = 0.047–0.001). The genetic risk score that combined the three loci also significantly interacted with *S. mutans* (*P* < 0.000 1). Functional analyses indicated that the identified genes are involved in the host immune response, galactose carbohydrate metabolism, and food-rewarding system, which could potentially be used to identify children at high risk for caries and to develop personalized caries prevention strategies.

## Introduction

Although largely preventable, dental caries remains the single most common chronic childhood disease, with nearly 1.8 billion new cases per year globally.^1–3^ Dental caries is a chronic infectious disease initiated from the virulent dental biofilms/plaque formed on tooth surfaces.^4^ Within the dental biofilms/plaque, oral cariogenic bacteria metabolize dietary carbohydrates, produce acid, and initiate demineralization of the tooth enamel.^5^ Although an enamel remineralization process takes place when the enamel is exposed to salivary calcium, phosphate, and fluoride ions, when the demineralization exceeds the remineralization process, dental caries occur.^6^

Traditional microbial risk markers for caries include *Streptococcus mutans*, a well-known culprit for dental caries, due to its acidogenicity, aciduricity, and capability of synthesizing dental plaque extracellular matrix.^7–11^ In theory, higher *S. mutans* carriage indicates higher caries risk. Interestingly, studies have identified a group of children who are colonized by *S. mutans* but do not manifest the disease, even with similar social–demographic–hygiene behavior characteristics compared with those of their counterparts.^12^ This phenomenon suggests that host susceptibility potentially plays a role in modulating the risk of *S. mutans* on the development of dental caries. Therefore, elucidating the interaction between host susceptibility, *S. mutans* carriage and their impact on caries onset could help identify high-risk child populations, and develop personalized precision dental caries prevention strategies.

It is speculated that host susceptibility to dental caries is influenced by each individual’s genetic constitution, including the tooth anatomy (deep groove and fissure), tooth hard tissue (enamel and dentin) quality, salivary properties, host immunity, oral microbiome composition, and taste preference,^13^ and the interactions between host gene and environmental factors, e.g., fluoride exposure.^14^ Recent genome-wide studies have reported novel associations of common genetic variants and gene-by-environment interactions with dental caries,^15–18^ such as the genes *TUFT1*, *NAMPT*, and *BMP7*, which are involved in enamel matrix and tooth development. The *NAMPT* gene (rs190395159) and the *BMP7* gene (rs72626594) were found to be associated with dental caries in a Hispanic/Latino community.^17^ In addition, an intergenic locus on chromosome 4q32 (rs4690994) was found to be strongly associated with early childhood caries.^15^ Zeng et al.^19^ reported that *BCOR*, *BCORL1, INHBA, CXCR1*, and *CXCR2* were associated with caries in permanent dentition adjusted for the presence of *S. mutans*.

However, given the bacterial infectious nature of dental caries, previous researchers have not examined the association between host genetics, oral microorganisms, and the onset of dental caries. Therefore, in this study, we hypothesized that certain host genes modulate host susceptibility to *S. mutans* in developing dental caries. To test this hypothesis, we investigated the influence of host gene-by-*S. mutans* interaction on dental caries in 709 children younger than 13 years of age in the United States.

## Results

### Participants’ characteristics

**Fig. 1:**
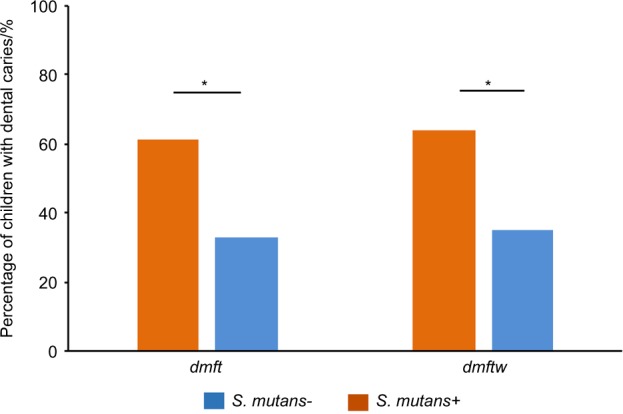
The relationship between dental caries and *S. mutans* carriage. *Indicates *P* *<* 0.05*. S. mutans*+ means positive for *S. mutans* carriage. *S. mutans*– means negative for *S. mutans* carriage

Genome-wide association analyses were conducted in 709 US children (<13 years old) using a dbGap database acquired from the center for oral health research in appalachia (COHRA) and the Iowa Head Start programmes (GEIRS). The participants’ characteristics included in this study are shown in Table 1. The majority of the participants were white (84%) and non-Hispanic (97%). There were slightly more male participants than females (52% vs. 48%). The prevalence of dental caries (*dmft and dmftw*) was 48% and 50%, respectively. *S. mutans* was detected in 53% of children. The mean age of children without *S. mutans* was lower than that of children with *S. mutans* (5.7 ± 3.0 vs. 7.1 ± 2.9 years of age, *P* < 0.000 1). *S. mutans* carriage was strongly associated with dental caries analyzed using the generalized estimating equation (GEE) and adjusting for covariates (*P* < 0.000 1). The prevalence of dental caries (*dmft and dmftw*) in children with *S. mutans* was nearly twice that in children without *S. mutans* (61% vs. 33%, *P* < 0.000 1) (Fig. 1).

**Table 1:** Demographic characteristics and dental caries outcomes of the participants (*n* = 709)

Characteristics *n* /%	Total (*n* = 709)	*S. mutans* + (*n* = 377)	*S. mutans* – (*n* = 332)	*P*-value (comparison between *S. mutans* + and –)
*Dental caries*				
*dmft* ≥ 1	340 (48%)	231 (61%)	109 (33%)	<0.0001
*dmftw* ≥ 1	355 (50%)	240 (64%)	115 (35%)	<0.0001
Age/year, mean (SD)	6.4 (3.0)	7.1 (2.9)	5.7 (3.0)	<0.0001
Male	371 (52%)	203 (54%)	168 (51%)	0.218
Race_White	587 (84%)	312 (84%)	275 (84%)	0.767
Ethnicity_Hispanic	22 (3%)	15 (4%)	7 (2%)	0.221

SD is the standard deviation. The *P* values were estimated by comparing children with or without *S. mutans* using ANOVA in 526 children randomly selected from each family. The average age of the *dmft* = 0 group and *dmft* ≥ 1 group was 5.6 ± 3.2 and 7.3 ± 2.6, respectively (*P* < 0.0001)

### Single-nucleotide polymorphisms associated with host caries susceptibility modified by *S. mutans* carriage

**Fig. 2:**
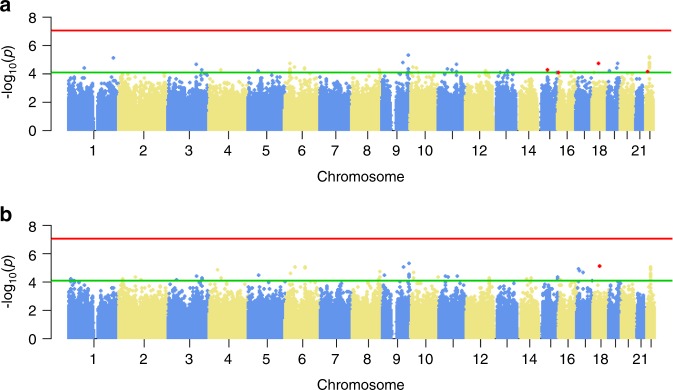
Genome-wide SNP-by-*S. mutans* interaction analysis results. **a** Manhattan plot for *dmft.* **b** Manhattan plot for *dmftw*. In both subfigures, the red line represents the genome-wide significant level; the green line represents the suggestive significance level; the red dots represent the SNPs identified in the study

Three loci (rs4786370, rs11635005, and rs1539849) interacted with *S. mutans* carriage and caries severity at a suggestive significance level among COHRA children (*P* < 9 × 10^−5^), and at a nominal significant level among GEIRS children (*P* = 0.047–0.001). At the discovery stage, genome-wide gene-by-*S. mutans* interaction analyses were performed. Manhattan plots are shown in Fig. 2. No interactions met the genome-wide significance level after Bonferroni correction (*P* < 9 × 10^−8^). The interactions with the lowest significance levels were between rs1831292 and *S. mutans* for both *dmft* and *dmftw* (*P* = 5.2 × 10^−6^ and 5.05 × 10^−6^, respectively). A relatively lenient significance value (*P* < 9 × 10^−5^) was then used to select the potential suggestive loci. A total of 53 single-nucleotide polymorphisms (SNPs) for *dmft* and *dmftw* were selected for replication.

At the replication stage, the conservative significance level was determined by Bonferroni correction for 53 assessed loci (*P* < 9.4 × 10^−4^). For *dmft*, the interaction between rs4786370 and *S. mutans* was close to the significance level, which had a *P*-value of 1 × 10^−3^ (Table 2). Three additional SNPs, rs11635005, rs6004787, and rs1539849 were significant at the nominal level (*P* < 0.05). For *dmftw*, one SNP, rs1539849 was significant at the nominal level. These four SNPs are located on chromosomes 15, 16, 18, and 22, respectively. The minor allele frequency ranged from 0.2 to 0.42. Allele frequencies of the four SNPs in the COHRA and GEIRS sites are shown in Appendix Table 1. The interactive effects between the four SNPs and *S. mutans* were consistent with those at the discovery stage (Table 2). The marginal effects of these SNPs, except for rs6004787 modified by *S. mutans* carriage status, were also concordant with those at the discovery stage (Appendix Fig. 1).

**Table 2:** Candidate genes and related dental caries outcomes

Outcome	SNP	CHR	EA	Location	EAF	*P* total	Coeff total	*P* discovery	Coeff discovery	*P* validation	Coeff validation	Gene
*Dmft*	rs11635005	15	T	Intronic	0.2	0.0001	−0.7	5.2 × 10^−5^	−1.33	0.004 8	−1.11	*GALK2*
*Dmft*	rs4786370	16	C	Upstream 2 kb	0.42	<0.0001	0.76	8.3 × 10^−5^	1.06	0.001	1.15	*IL32*
*Dmft*	rs1539849	18	C	Intronic	0.7	0.0001	−0.63	1.9 × 10^−5^	−1.28	0.046 8	−0.82	*CELF4*
*Dmftw*	rs1539849	18	C	Intronic	0.7	0.0001	−0.63	8 × 10^−6^	−1.32	0.046 8	−0.82	*CELF4*

Total means the value obtained from the entire sample. Discovery means the value obtained at the discovery stage. Validation means the value obtained at the validation stage

CHR, chromosome; EA, effective allele; EAF, effective allele frequency; Coeff, coefficient of the interaction

**Fig. 3:**
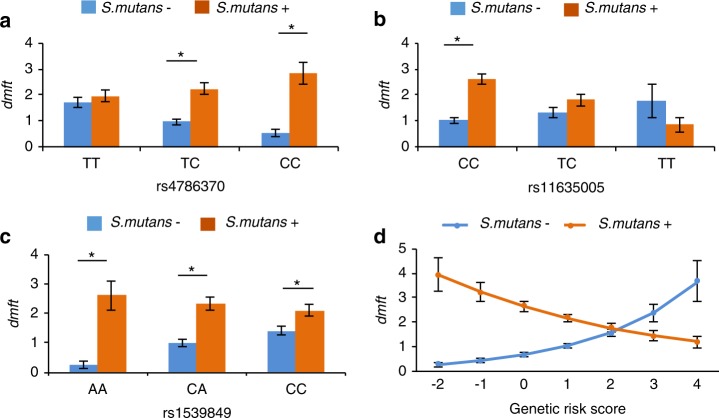
Marginal effect of SNP-by-*S. mutans* interaction on caries *(dmft)*. The marginal effect was estimated using the GEE negative binomial models adjusting for age, gender, race, and five eigenvectors. *P < 0.05. **a** Marginal effect of rs4786370-by-*S. mutans* interaction on *dmft.* **b** Marginal effect of rs11635005-by-*S. mutans* interaction on *dmft*. **c** Marginal effect of rs1539849-by-*S. mutans* interaction on *dmft* **d** Marginal effect of the GRS on *dmft*

Caries severity (*dmft*) was dependent on individual genotypes and *S. mutans* carriage. The effect of the three gene-by-*S. mutans* interactions on *dmft* is presented in Fig. 3. The effect of rs1539849-by-*S. mutans* on *dmftw* was similar to its effect on *dmft* (data not shown). For rs4786370 (Fig. 3a), *S. mutans* was significantly associated with dental caries severity when children carried the TC and CC alleles (*P* < 0.05). For rs11635005 (Fig. 3b), *S. mutans* was significantly associated with dental caries severity when children carried the CC alleles (*P* < 0.05). For rs1539849 (Fig. 3c), regardless of alleles, *S. mutans* was significantly associated with caries susceptibility (*P* < 0.05). The difference in caries severity affected by *S. mutans* carriage was more prominent among children, who carried the AA and CA alleles compared with children with CC alleles. When the marginal effects of the three SNPs in the whole sample were compared with those in the COHRA and GEIRS sites separately, the effects were consistent between the two sites and the whole sample (Fig. 3 and Appendix Fig. 1).

When the caries severity was compared in children with or without *S. mutans*, children with varied genotypes of rs11635005 had significant differences in caries severity when *S. mutans* was present (pairwise comparison *P* < 0.05). Children with varied genotypes of rs4786370 or rs1539849 had significant differences in caries severity when *S. mutans* was absent (pairwise comparison *P* < 0.05).

The joint effect of the three SNPs, rs4786370, rs11635005, and rs1539849 was calculated as the genetic risk score (GRS), which ranged from −2 to 4. The interaction between the GRS and *S. mutans* was significant in the whole sample and the two sites (*P* < 0.000 1). The marginal effect of the GRS is presented in Fig. 3d. For children without *S. mutans* carriage, the risk for dental caries increased when the GRS increased, whereas for children with *S. mutans* carriage, the risk of dental caries decreased with increasing GRS.

The results of sensitivity analyses in white children and the subsample with the assessment of home water source are presented in Table 3. The effect sizes of the three SNPs in sensitivity analyses were similar to those in the whole sample. All interactions between SNPs and *S. mutans* were significant (*P* < 0.05).

**Table 3:** Sensitivity analysis of the three identified SNPs

Outcome	SNP	CHR	EA	Coeff_White	P_White	Coeff_Watersource	P_Watersource	Gene
*Dmft*	rs11635005	15	T	−0.74	0.002	−0.72	0.0016	GALK2
*Dmft*	rs4786370	16	C	0.73	0.0008	0.75	0.001	IL32
*Dmft*	rs1539849	18	C	−0.56	0.0124	−0.64	0.0055	CELF4

CHR is chromosome. EA is effective allele. Coeff is the coefficient of the interaction. Results were obtained using the GEE model with a negative binomial distribution and adjusting for age, sex, race (not adjusted in the analysis with white children), site, and home water source (only adjusted in the analysis with the subsample with the information about water source)

### Functional analysis of the identified SNPs

**Fig. 4:**
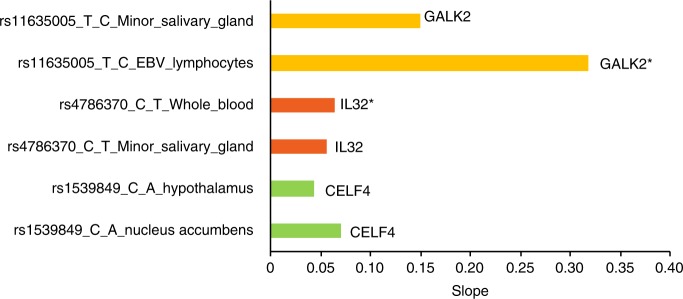
The impact of effective alleles on gene expression. The labels were organized as SNP_effective allele_reference allele_tissue. The slope values were obtained from SNP-gene association tests conducted by GTEx. A positive slope value indicates that the effect allele is associated with increased gene expression. *P < 0.05

Functional analysis of the three SNPs and SNPs in linkage disequilibrium (LD; *R*^2^ ≥ 0.8) was conducted using HaploReg. Two SNPs, rs11635005 and rs1539849, are located within intron regions of the *galactokinase 2* (*GALK2)* and *CUGBP, Elav-like family member 4* (*CELF4)* genes, and rs4786370 is located within 2 kb upstream of the *interleukin 32* (*IL32)* gene. Although no SNPs are located within exons (protein-coding regions), all identified SNPs and SNPs in LD are located at the promoter/enhancer histone markers and/or DNAse hypersensitive regions (Appendix Table 2), which means that these SNPs or SNPs in LD may affect the expression of relevant genes. Two SNPs, rs11635005 and rs4786370, have been identified as the expression quantitative trait loci that affect the expression levels of mRNAs. Moreover, analysis of the genes listed in Table 2 that harbor these SNPs using GTEx showed that *GALK2* is ubiquitously expressed (Appendix Fig. 2). *IL32* is highly expressed in the blood and gastrointestinal organs. *CELF4* is mainly expressed in the brain. The impact of the effective alleles on annotated genes was assessed in expression-specific tissues and the minor salivary gland (Fig. 4). The effective alleles of rs11635005 and rs4786370 are significantly associated with increased expression of *GALK2* in the EBV-transformed lymphocytes, and *IL32* in the whole blood, respectively. These alleles have similar but nonsignificant effects on their annotated genes in the minor salivary glands. The effective alleles of rs1539849 are not associated with expression changes in their annotated genes.

Ontological analysis of the three annotated genes using Enrichr and ToppFun indicated that (1) *GALK2* is involved in the galactose metabolic process and carbohydrate phosphorylation (Appendix Fig. 3); (2) *IL32* participates in the cytokine-mediated signaling pathway and is associated with multiple inflammatory diseases (Appendix Table 3). *CELF4* is related to the modulation of excitatory postsynaptic potential and regulation of mRNA splicing. *CELF4* is significantly expressed in the nucleus accumbens, both in humans and mice, which is the center of the food reward circuitry. In addition, *CELF4* is linked to an increased susceptibility to weight gain (*P* = 0.001 5) (Appendix Fig. 4).

## Discussion

Our results indicate that dental caries severity in the primary dentition was potentially related to individual genotypes and *S. mutans* carriage. Although no gene-by-*S. mutans* interactions met the strict genome-wide significance level, we identified three potential SNPs, rs4786370, rs11635005, and rs1539849, that interacted with *S. mutans* at the suggestive significance level (*P* < 9 × 10^−5^). These interactions were replicated at the nominal level in an independent cohort. We adopted the approach used in previous GWAS with a relatively lenient *p*-value to select suggestive genetic loci for replication.^15,18^ In the replication step, we used the conservative significance level and nominal significance level, which was used in a previous genome-wide study.^18^ These SNPs were not identified by previous genome-wide studies, because we assessed gene-by-environment interactions instead of direct genetic associations, and previous studies have shown that novel genes could be identified by examining the modification effect of environmental factors on genetic associations.^14,20^ The severity of dental caries was associated with individual host genotypes and *S. mutans* carriage. For instance, dental caries severity was significantly influenced by *S. mutans* carriage in children who carried CC alleles but not TC or TT alleles at rs11635005. In addition to the individual SNP, the GRS that combined the effect of these three SNPs significantly interacted with *S. mutans* carriage. Further ontological analysis of the annotated genes for the identified SNPs suggests that *IL32*, *GALK2*, and *CELF4* are potentially plausible genes that may play a role in the development of dental caries and interact with *S. mutans* through their involvement in galactose and carbohydrate metabolism, host immune response, and food consumption.

### *IL32*

The SNP rs4786370 is mapped to *IL32*, which encodes a member of the proinflammatory cytokine family.^21^ *IL32* has nine isoforms and regulates the expression of numerous inflammatory cytokines, such as TNF-α, IL1β, IL6, and IL10. Some of these cytokines are elevated in saliva, in children with dental caries.^22,23^ Furthermore, *IL32* has been associated with various infectious/inflammatory diseases,^24–27^ including periodontitis.^28^

In this study, we found that children with the C allele of rs4786370 were associated with increased caries severity in the presence of *S. mutans*. When *S. mutans* was absent, dental caries severity was significantly different among children with varied genotypes of rs4786370. Children who carried the C allele presented with lower caries severity when *S. mutans* was absent. This phenomenon is possibly because the C allele at rs4786370 is associated with higher expression of *IL32*. When *S. mutans* is absent, higher *IL32* expression may indicate an enhanced immune response and better host protection from dental caries.

### *GALK2*

The SNP rs11635005 is located within *GALK2*. *GALK2* encodes an N-acetylgalactosamine (GalNAc) kinase. This enzyme can efficiently phosphorylate GalNAc at low concentrations and phosphorylate galactose at high concentrations.^29^ The activity of GalNAc kinase is pH-dependent, with the highest activity at pH 7–8.^30^ GalNAc and galactose are monosaccharides (simple sugar). GalNAc is found in a variety of human glycoproteins that participate in numerous biological processes, including immune response^31^ and tumor cell surface expression.^32^ Galactose is often found in dairy products, which form lactose with glucose.^33^

In the oral cavity, GalNAc and galactose could be metabolized through two paths, including phosphorylation by human enzymes, such as GalNAc kinase encoded by *GALK2*, and metabolism by acidogenic oral bacteria, including *S. mutans*, *S. salivarius*, and *Lactobacillus*.^34^ The bacteria-involved galactose metabolism is associated with more acid production and lower plaque pH. Our study results showed that for children who have CC alleles at rs11635005, the presence of *S. mutans* significantly increased caries severity. This phenomenon can be explained as follows: the C allele of rs11635005 is associated with lower expression of *GALK2*, which manifested as attenuated GalNAc kinase-involved galactose metabolism; without *S. mutans carriage*, the caries severity among children with different *GALK2* genotypes was not significantly different. However, with *S. mutans carriage*, while children who carry CC alleles may have lower host enzyme activity to metabolize galactose, the bacteria-involved galactose metabolism may be enhanced. Therefore, more acid production and lower plaque pH increase caries risk among children with CC alleles.

In contrast, for children with the T allele of rs11635005, the carriage of oral *S. mutans* was not significantly associated with caries severity. The T allele is associated with higher expression of *GALK2* and higher production of GalNAc kinase. It is speculated that with sufficient GalNAc kinase-involved galactose metabolism, bacteria-involved galactose metabolism is not significantly increased with the presence of *S. mutans*. Therefore, children with the T allele are not susceptible to *S. mutans* carriage in relation to caries risk.

### *CELF4*

The SNP rs1539849 is located at an intron region of *CELF4*. *CELF4* is predominantly expressed in the brain. It plays a role in maintaining the stability and availability of mRNA for numerous proteins in excitatory neurons.^35^ Consequently, *CELF4* regulates synaptic plasticity and transmission. Other than epilepsy, *CELF4* mutant mice also presented hyperactivity and late-onset obesity.^36^ This obesity trait was possibly due to increased food intake rather than reduced energy expenditure, as *CELF4* mutant mice were hyperactive. Furthermore, *CELF4* is highly expressed in the nucleus accumbens, the center of food reward circuitry that is linked to overeating and palatable food intake.^37,38^ Therefore, *CELF4* may play a role in the regulation of dietary intake.

In this study, *CELF4* was found to be associated with *S. mutans* carriage and dental caries risk in primary dentition. The dental caries severity was significantly different among children with varied genotypes at rs1539849, when *S. mutans* was absent. *CELF4* might not have a direct relationship with *S. mutans* carriage or its pathogenicity. However, children with certain genotypes at variants within *CELF4*, such as rs1539849, might be at higher risk for overeating, particularly sweetened food consumption, and consequently have an increased risk for dental caries.

The findings from this study need to be cautiously interpreted with the following considerations: (1) the limited sample size, which is potentially the reason that no genome-wide significant interactions were identified; (2) the limited microbiological data. Only binary (yes/no) data of *S. mutans* assessments were available, and we were not able to assess the impact of the oral carriage scale of *S. mutans*. In addition, the detection of oral *S. mutans* depends on clinical sample types (swab, saliva, or plaque samples; carious or non-carious lesions) and *S. mutans* identification methods (culture-dependent or culture-independent). This critical information was not provided in the original dataset. Furthermore, there are no microbiological data on other cariogenic or beneficial microorganisms. For instance, *Candida* species and *Lactobacillus* species have been shown to be associated with caries in children;^39,40^ *Streptococcus salivarius, Streptococcus sobrinus*, *Streptococcus parasanguinis, Streptococcus wiggsiae*, *Streptococcus exigua, Parascardovia denticolens, Porphyromonas, Actinomyces*, and *Veillonella* have been identified in the oral microbiota of caries-active children in addition to *S. mutans*;^41–46^ the role of these cariogenic or beneficial microorganisms in this hypothesized host gene-by-*S. mutans* interaction on dental caries could not be tested using the current dataset; (3) the lack of data on other environmental factors associated with dental caries and *S. mutans*, such as water fluoridation (only 48% of the participants had data on home fluoride exposure level), oral hygiene, salivary flow, socioeconomic status, and dietary intake; and (4) the limited portion of racially and ethnically diverse participants, which precludes the generalization of our findings to nonwhite and Hispanic children. Therefore, future studies with a larger, more diverse population and additional caries risk factors are desirable to validate the current findings.

To summarize, we identified three human genes that are likely associated with the interaction between the host, *S. mutans*, and dental caries in children. These human genes are involved in the host immune response, galactose carbohydrate metabolism, and potential food-rewarding system. Further validation of these candidate genes in the development of dental caries in children is warranted. Given that additional oral cariogenic microorganisms have been identified in addition to *S. mutans*, further research should consider investigating the relationship of these genes with the oral microbiota and the development of dental caries. The integration of human genome and oral microbiome findings in dental caries risk assessment in children could be used to develop more effective, personalized, predictive, and preventive strategies for dental caries.

## Materials and methods

### Study population

This study was approved by the University of Rochester Research Subject Review Board. The assessed dataset, Dental Caries: Whole Genome Association and Gene×Environment Studies, was obtained from the NIH Database of Genotypes and Phenotypes (https://www.ncbi.nlm.nih.gov/gap). The study subjects in this dataset were recruited from four sites at the University of Pittsburgh and the University of Iowa: (1) the COHRA, (2) the Dental Registry and DNA Repository (DRDR), (3) the Iowa Fluoride Study (IFS), and (4) the GEIRS.^18,47^ Study subject recruitment and data collection were detailed previously.^18,47^

The dataset obtained from dbGaP included 5 418 participants with dental phenotype data. In this study, participants who met the following inclusion criteria were selected: (a) younger than 13 years of age; (b) having *S. mutans* data; (c) having records of dental caries in primary dentition; and (d) having genotyping data. In total, 709 children from 526 families (COHRA and GEIRS sites) were eligible for the gene-by-*S. mutans* interaction analysis. Two-stage analysis (discovery and replication) was performed to identify potential gene-by-*S. mutans* interactions. The data from the COHRA site (*n* = 547) were used to conduct the genome-wide analysis at the discovery stage. The data from the GEIRS site (*n* = 162) were used at the replication stage to assess the findings identified at the discovery stage.

### Phenotypes and covariates

Dental caries diagnosis was detailed by Shaffer et al.^18^ Upon intra-oral examination, each primary tooth was scored as sound (no white spot or cavitated lesion), white spots (non-cavitated lesion), decayed (cavitated lesion), or missing/filled due to caries. Based on these scorings, two indices were calculated: *dmft* including decayed (d) missing due to decay (m), filled (f), and teeth (t) and *dmftw* (including both *dmft* and white spots). *S. mutans* detection status was coded as “yes/no” in the original study. Demographic characteristics that were marginally associated with primary dental caries (*P* < 0.2) were included as covariates, which were age, gender, and race (white/nonwhite).

### Genotyping methods

DNA samples were extracted from blood, saliva, buccal mucosal swabs, or mouthwash. A custom panel of 580 000 SNPs was genotyped using the Illumina 610 platform (Illumina, Inc., San Diego, CA, USA). In this study, SNPs were filtered based on the following exclusion criteria: (a) located on autosomal chromosomes; (b) genotyping call rate <95%; (c) minor allele frequency <1%; and (d) Hardy–Weinberg equilibrium *P* value < 5 × 10^−7^. In total, 556 839 SNPs passed the quality assurance criteria and were used for the genome-wide analyses.

### Statistical analysis

The population substructure was assessed using principal component analysis in PLINK1.9 (https://www.cog-genomics.org/plink2).^48,49^ The first five eigenvectors were generated and adjusted as covariates. At the discovery stage, the genome-wide gene-by-*S. mutans* interaction analyses were conducted using the GWAF package in R, which utilizes GEE to adjust for family clusters.^50^ Binomial distribution of the dichotomized dental caries outcomes (*dmft* and *dmftw*) was applied. An additive genetic model with the number of effective alleles in a SNP treated as a numeric variable was used in the analyses. The interactions between SNPs and *S. mutans* were assessed by including a product term ($x_{\mathrm{additive}} \times x_{{S.mutans}}$) in the regression models.^50,51^ To maximize sample size and power, stratified analyses were not conducted at the genome-wide association analysis stage.^52^

$$\begin{array}{l}{\mathrm{logit}}(p) = \beta _0 + \beta _1x_{\mathrm{additive}} + \beta _2x_{S.mutans}\\ + \ \beta _3x_{\mathrm{additive}} \times x_{S.mutans} + \beta _4x_{\mathrm{covariates}} + {\mathrm{\varepsilon }}\end{array}$$

The genome-wide *P* value was set at 9 × 10^−8^ after Bonferroni correction for the total number of SNPs tested in the analyses. The Bonferroni correction is a commonly used method to control multiple testing in GWAS.^53^ Age, gender, and race were controlled in the statistical models, although the ages were different among the *dmft* = 0 and *dmft* > = 1 groups.

At the replication stage, we assessed the potential suggestive SNPs selected at the discovery stage. Dental caries outcomes used as count data were analyzed using GEE, with a negative binomial distribution in STATA 15.0. Two significance levels were used. The conservative significance level was set based on Bonferroni correction, adjusting for the number of SNPs assessed. The nominal significance level was also used. Finally, the effects of gene-by-*S. mutans* interactions were estimated using GEE with a negative binomial distribution in the whole sample (*n* = 709).

The joint effect of the replicated SNPs represented as a GRS was calculated based on the effective alleles’ impact on *dmft* when *S. mutans* carriage was negative. If the effective allele of an SNP was associated with an increased risk of *dmft*, the GRS score of this SNP was coded as “0, + 1, + 2” based on the number of the effective allele. If the effective allele of a SNP was associated with a decreased risk of *dmft*, the GRS score of the SNP was coded as “0, −1, −2” based on the number of the effective allele. The interactive effect of GRS with *S. mutans* was estimated using GEE with a negative binomial distribution.

We also conducted sensitivity analyses to evaluate the robustness of our identified SNPs using the GEE model with a negative binomial distribution in STATA 15.0. The top SNPs were assessed in white children (*n* = 587). Nonwhite children were not assessed due to limited sample size. We also assessed the top SNPs in a subsample containing an assessment of home water source (*n* = 509). Home water source was classified as water from the city/public, well, or others.

The functional annotation of the identified SNPs was conducted using HaploReg v4.1^54^ and GTEx Portal v7. Functional inference of the genes harboring the identified SNPs was conducted using Enrichr^55^ and ToppFun.^56^ This study was conducted following the STREGA reporting guidelines.^57^

## Conclusions

This study identified three gene-by-*S. mutans* interactions that were potentially associated with the severity of dental caries in the primary dentition with plausible biological roles. These genetic markers were linked to varied caries risk modified by *S. mutans* carriage status. Our study findings could help to elucidate the relationship between host susceptibility, *S. mutans* carriage, and caries development. The identified human genes could be used to identify children at high risk of caries and develop personalized dental caries prevention strategies. The identified genes could also serve as potential targets for caries prevention.

## Supplementary information

Supplement material

## Data Availability

The datasets used for the analyses described in this paper were obtained from the NIH Database of Genotypes and Phenotypes through dbGaP accession number phs000095.v3.p1. The study, Dental Caries: Whole Genome Association and Gene × Environment Studies, was funded by the NIDCR, grant number U01-DE018903. https://www.ncbi.nlm.nih.gov/projects/gap/cgi-bin/study.cgi?study_id=phs000095.v3.p1.
