# Supplementary material for: Human genes influence the interaction between Streptococcus mutans and host caries susceptibility: a genome-wide association study in children with primary dentition
Source: Int J Oral Sci. 2019 May 30;11(2):19. doi: 10.1038/s41368-019-0051-4 (PMC6544625; doi:10.1038/s41368-019-0051-4)
Supplement: Supplementary file 1 — Supplement material [file 41368_2019_51_MOESM1_ESM.docx]

**Appendix**

**Human genes influence the interaction between *Streptococcus mutans* and host caries susceptibility: a genome-wide association study in children with primary dentition**

Ying Meng^1^, Tong Tong Wu^2^, Ronald Billings^3^, Dorota T. Kopycka-Kedzierawski ^3^, Jin Xiao^3*^

^1^ School of Nursing, University of Rochester, Rochester, NY, USA

^2^ Department of Biostatistics and Computational Biology, University of Rochester, NY, USA

^3^ Eastman Institute for Oral Health, University of Rochester Medical Center, Rochester, NY, USA

*corresponding author

**Appendix Figure 1. Marginal effect of SNP-by-*S. mutans* interaction on caries *(dmft)* in two sites.**

Marginal effect was estimated using the generalized estimating equation negative binomial models adjusting for age, gender, race, and 5 eigenvectors.

**Appendix Figure 2. Tissue-specific expression profiles for genes harboring the identified SNPs.**

Gene expression data and figures were obtained from GTEx portal. TPM means transcripts per million. Each bar represents the expression level of the gene at the specified tissue.

|  |
| --- |

**Appendix Figure 3. GO Biological process enrichment for *GALK2***

FDR represents false discovery rate. FDR results were obtained from TopFunn using data from Gene Ontology Consortium.

|  |
| --- |

**Appendix Figure 4. Mammalian phenotype enrichment for *CELF4***

Adj. p value represents adjusted p value, which is corrected p value using the Benjamini-Hochberg method in Enrichr.

**Appendix Table 1. Allele frequency among two datasets**

| *S. mutans* | Site= COHRA | | | Site= GEIRS | | |
| --- | --- | --- | --- | --- | --- | --- |
|  | rs4786370 | | | | | |
|  | TT | TC | CC | TT | TC | CC |
| NO | 86 | 126 | 39 | 25 | 42 | 13 |
| Yes | 101 | 143 | 50 | 32 | 28 | 22 |
|  | rs11635005 | | | | | |
|  | CC | TC | TT | CC | TC | TT |
| NO | 164 | 80 | 8 | 53 | 22 | 5 |
| Yes | 179 | 103 | 12 | 54 | 21 | 7 |
|  | rs6004787 | | | | | |
|  | CC | TC | TT | CC | TC | TT |
| NO | 139 | 102 | 9 | 54 | 25 | 1 |
| Yes | 175 | 97 | 22 | 42 | 33 | 7 |
|  | rs1539849 | | | | | |
|  | AA | CA | CC | AA | CA | CC |
| NO | 18 | 97 | 137 | 4 | 42 | 34 |
| Yes | 39 | 120 | 135 | 3 | 37 | 42 |

*****COHRA: The Center for Oral Health Research in Appalachia study; GEIRS: The Iowa Head Start study

**Appendix Table 2. Functional annotation of the three identified SNPs and SNPs in linkage disequilibrium**

| chr | query_snp_rsid | rsID | D' | ref | alt | AMR | Chromatin_States | Chromatin_States_Imputed | Chromatin_Marks | DNAse | Proteins | eQTL | grasp | Motifs | gene | region |
| --- | --- | --- | --- | --- | --- | --- | --- | --- | --- | --- | --- | --- | --- | --- | --- | --- |
| 15 | rs11635005 | rs113467140 | 0.92 | A | G | 0.17 |  |  | E050,H3K27ac_Enh |  | . | yes | . | Ik-1_2; Maf_disc2; NRSF_disc10; Sin3Ak-20_disc1 | GALK2 | intronic |
| 15 | rs11635005 | rs113804499 | 0.92 | G | T | 0.17 |  |  | E025,H3K4me3_Pro;E078,H3K4me3_Pro;E083,H3K4me3_Pro;E110,H3K4me3_Pro |  | . | yes | . | AIRE_1;Evi-1_4; Irf_known10;Irf_known11;Mef2_known6;NF-AT;SRF_known5;STAT_disc3;TATA_known4;TFIIA | GALK2 | intronic |
| 15 | rs11635005 | rs78298181 | 0.92 | T | A | 0.17 | E061,7_Enh |  | E061,H3K4me1_Enh;E077,H3K4me1_Enh;E109,H3K4me1_Enh |  | . | yes | . | Dbx1;HDAC2_disc6;Pou2f2_known8;TATA_known1;Zfp105 | GALK2 | intronic |
| 15 | rs11635005 | rs73392236 | 0.94 | C | A | 0.17 | E006,7_Enh;E027,7_Enh;E028,7_Enh;E031,7_Enh;E032,7_Enh;E046,7_Enh;E083,7_Enh;E094,7_Enh;E095,7_Enh;E096,7_Enh;E110,7_Enh;E116,7_Enh |  | E001,H3K4me1_Enh;E006,H3K4me1_Enh;E018,H3K4me1_Enh;E024,H3K4me1_Enh;E027,H3K4me1_Enh;E028,H3K4me1_Enh;E031,H3K4me1_Enh;E032,H3K4me1_Enh;E034,H3K4me1_Enh;E038,H3K4me1_Enh;E043,H3K4me1_Enh;E046,H3K4me1_Enh;E047,H3K4me1_Enh;E054,H3K4me1_Enh;E058,H3K4me1_Enh;E059,H3K4me1_Enh;E062,H3K4me1_Enh;E077,H3K4me1_Enh;E083,H3K4me1_Enh;E094,H3K4me1_Enh;E095,H3K4me1_Enh;E096,H3K4me1_Enh;E098,H3K4me1_Enh;E100,H3K4me1_Enh;E104,H3K4me1_Enh;E105,H3K4me1_Enh;E107,H3K4me1_Enh;E108,H3K4me1_Enh;E109,H3K4me1_Enh;E110,H3K4me1_Enh;E113,H3K4me1_Enh;E116,H3K4me1_Enh;E006,H3K9ac_Pro;E027,H3K9ac_Pro;E062,H3K9ac_Pro;E124,H3K9ac_Pro;E012,H3K27ac_Enh;E056,H3K27ac_Enh;E059,H3K27ac_Enh;E061,H3K27ac_Enh;E117,H3K27ac_Enh;E124,H3K27ac_Enh;E076,H3K4me3_Pro | E083 | . | yes | . | CAC-binding-protein;INSM1;MAZ;MZF1::1-4_2;Pou2f2_disc2;SP1_disc3;SP1_known1;STAT_disc7;TFII-I;UF1H3BETA;VDR_2;WT1;Zfp281 | GALK2 | intronic |
| 15 | rs11635005 | rs76231511 | 0.94 | G | T | 0.15 |  |  | E061,H3K4me1_Enh;E124,H3K27ac_Enh |  | . | yes | . | FAC1;Fox;Foxa_known1;Foxa_known2;Foxa_known4;Foxd3;Foxi1;Foxj1_1;Foxj1_2;Foxj2_1;Foxo_1;Foxp1;HDAC2_disc2;Sox_6;TATA_known4;Zfp105;p300_disc3 | GALK2 | intronic |
| 15 | rs11635005 | rs73392249 | 0.94 | G | A | 0.17 |  |  | E014,H3K27ac_Enh;E124,H3K27ac_Enh;E014,H3K4me1_Enh;E015,H3K4me1_Enh;E018,H3K4me1_Enh;E019,H3K4me1_Enh;E037,H3K4me1_Enh;E040,H3K4me1_Enh;E041,H3K4me1_Enh;E042,H3K4me1_Enh;E047,H3K4me1_Enh;E055,H3K4me1_Enh;E056,H3K4me1_Enh;E061,H3K4me1_Enh;E016,H3K9ac_Pro;E067,H3K4me3_Pro;E078,H3K4me3_Pro | E123 | . | yes | . | Cart1;Foxa_disc2;Foxa_known4;Foxp1;HNF1_7;Pou1f1_1;Pou2f2_known5;Pou2f2_known8;Pou3f3;Pou5f1_disc2;Pou6f1_1 | GALK2 | intronic |
| 15 | rs11635005 | rs73392256 | 0.94 | T | A | 0.15 |  |  | E040,H3K4me1_Enh;E041,H3K4me1_Enh;E047,H3K4me1_Enh;E048,H3K4me1_Enh;E110,H3K4me1_Enh;E078,H3K4me3_Pro;E088,H3K4me3_Pro;E124,H3K27ac_Enh |  | . | yes | . | Pou5f1_disc1 | GALK2 | intronic |
| 15 | rs11635005 | rs117582142 | 0.94 | C | T | 0.15 |  |  | E041,H3K4me1_Enh;E047,H3K4me1_Enh;E048,H3K4me1_Enh;E110,H3K4me1_Enh;E078,H3K4me3_Pro;E088,H3K4me3_Pro;E124,H3K27ac_Enh |  | . | yes | . | GATA_known8;HDAC2_disc6 | GALK2 | intronic |
| 15 | rs11635005 | rs11638515 | 0.94 | C | T | 0.15 | E030,7_Enh;E083,7_Enh | E083,22_PromP;E124,17_EnhW2 | E001,H3K4me3_Pro;E015,H3K4me3_Pro;E020,H3K4me3_Pro;E124,H3K4me3_Pro;E014,H3K4me1_Enh;E018,H3K4me1_Enh;E019,H3K4me1_Enh;E020,H3K4me1_Enh;E030,H3K4me1_Enh;E059,H3K4me1_Enh;E061,H3K4me1_Enh;E083,H3K4me1_Enh;E101,H3K4me1_Enh;E105,H3K4me1_Enh;E114,H3K4me1_Enh;E124,H3K4me1_Enh;E016,H3K27ac_Enh;E069,H3K27ac_Enh;E071,H3K27ac_Enh;E072,H3K27ac_Enh;E124,H3K27ac_Enh;E129,H3K27ac_Enh;E025,H3K9ac_Pro;E027,H3K9ac_Pro;E062,H3K9ac_Pro;E083,H3K9ac_Pro;E101,H3K9ac_Pro;E124,H3K9ac_Pro;E125,H3K9ac_Pro |  | . | yes | . | Foxo_2;Sox_10;Sox_13;TCF4_known3 | GALK2 | intronic |
| 15 | rs11635005 | rs111790778 | 0.94 | G | A | 0.15 | E051,6_EnhG;E124,7_Enh | E029,17_EnhW2;E030,17_EnhW2;E124,18_EnhAc | E017,H3K27ac_Enh;E029,H3K27ac_Enh;E049,H3K27ac_Enh;E050,H3K27ac_Enh;E055,H3K27ac_Enh;E056,H3K27ac_Enh;E069,H3K27ac_Enh;E071,H3K27ac_Enh;E072,H3K27ac_Enh;E074,H3K27ac_Enh;E075,H3K27ac_Enh;E080,H3K27ac_Enh;E101,H3K27ac_Enh;E106,H3K27ac_Enh;E109,H3K27ac_Enh;E111,H3K27ac_Enh;E117,H3K27ac_Enh;E124,H3K27ac_Enh;E126,H3K27ac_Enh;E128,H3K27ac_Enh;E129,H3K27ac_Enh;E017,H3K9ac_Pro;E025,H3K9ac_Pro;E027,H3K9ac_Pro;E038,H3K9ac_Pro;E062,H3K9ac_Pro;E068,H3K9ac_Pro;E072,H3K9ac_Pro;E074,H3K9ac_Pro;E083,H3K9ac_Pro;E088,H3K9ac_Pro;E101,H3K9ac_Pro;E124,H3K9ac_Pro;E125,H3K9ac_Pro;E024,H3K4me1_Enh;E025,H3K4me1_Enh;E029,H3K4me1_Enh;E036,H3K4me1_Enh;E037,H3K4me1_Enh;E041,H3K4me1_Enh;E042,H3K4me1_Enh;E044,H3K4me1_Enh;E046,H3K4me1_Enh;E047,H3K4me1_Enh;E049,H3K4me1_Enh;E050,H3K4me1_Enh;E051,H3K4me1_Enh;E055,H3K4me1_Enh;E063,H3K4me1_Enh;E086,H3K4me1_Enh;E087,H3K4me1_Enh;E088,H3K4me1_Enh;E122,H3K4me1_Enh;E124,H3K4me1_Enh;E126,H3K4me1_Enh;E128,H3K4me1_Enh;E050,H3K4me3_Pro |  | . | yes | . | . | GALK2 | intronic |
| 15 | rs11635005 | rs11632303 | 0.94 | T | A | 0.17 | E014,7_Enh;E016,7_Enh;E018,7_Enh;E019,7_Enh;E020,7_Enh;E023,7_Enh;E025,7_Enh;E026,2_TssAFlnk;E030,7_Enh;E049,7_Enh;E050,7_Enh;E051,7_Enh;E067,7_Enh;E076,7_Enh;E083,7_Enh;E088,7_Enh;E092,7_Enh;E103,7_Enh;E111,7_Enh;E117,7_Enh;E128,7_Enh;E129,7_Enh | E001,17_EnhW2;E002,17_EnhW2;E003,17_EnhW2;E012,17_EnhW2;E014,17_EnhW2;E015,17_EnhW2;E016,17_EnhW2;E017,17_EnhW2;E018,17_EnhW2;E019,17_EnhW2;E020,17_EnhW2;E023,13_EnhA1;E025,13_EnhA1;E026,13_EnhA1;E030,18_EnhAc;E049,15_EnhAF;E052,15_EnhAF;E076,17_EnhW2;E078,17_EnhW2;E081,17_EnhW2;E083,14_EnhA2;E086,17_EnhW2;E088,15_EnhAF;E103,17_EnhW2;E111,17_EnhW2;E114,17_EnhW2;E117,18_EnhAc;E121,18_EnhAc;E125,18_EnhAc;E126,15_EnhAF;E129,15_EnhAF | E001,H3K4me1_Enh;E002,H3K4me1_Enh;E003,H3K4me1_Enh;E014,H3K4me1_Enh;E015,H3K4me1_Enh;E016,H3K4me1_Enh;E017,H3K4me1_Enh;E018,H3K4me1_Enh;E019,H3K4me1_Enh;E020,H3K4me1_Enh;E023,H3K4me1_Enh;E024,H3K4me1_Enh;E025,H3K4me1_Enh;E026,H3K4me1_Enh;E030,H3K4me1_Enh;E049,H3K4me1_Enh;E050,H3K4me1_Enh;E051,H3K4me1_Enh;E061,H3K4me1_Enh;E063,H3K4me1_Enh;E067,H3K4me1_Enh;E068,H3K4me1_Enh;E069,H3K4me1_Enh;E071,H3K4me1_Enh;E074,H3K4me1_Enh;E076,H3K4me1_Enh;E078,H3K4me1_Enh;E081,H3K4me1_Enh;E082,H3K4me1_Enh;E083,H3K4me1_Enh;E086,H3K4me1_Enh;E087,H3K4me1_Enh;E088,H3K4me1_Enh;E092,H3K4me1_Enh;E101,H3K4me1_Enh;E103,H3K4me1_Enh;E109,H3K4me1_Enh;E111,H3K4me1_Enh;E117,H3K4me1_Enh;E121,H3K4me1_Enh;E126,H3K4me1_Enh;E128,H3K4me1_Enh;E129,H3K4me1_Enh;E003,H3K27ac_Enh;E012,H3K27ac_Enh;E014,H3K27ac_Enh;E015,H3K27ac_Enh;E017,H3K27ac_Enh;E019,H3K27ac_Enh;E026,H3K27ac_Enh;E045,H3K27ac_Enh;E049,H3K27ac_Enh;E050,H3K27ac_Enh;E075,H3K27ac_Enh;E076,H3K27ac_Enh;E078,H3K27ac_Enh;E089,H3K27ac_Enh;E090,H3K27ac_Enh;E092,H3K27ac_Enh;E103,H3K27ac_Enh;E111,H3K27ac_Enh;E117,H3K27ac_Enh;E124,H3K27ac_Enh;E126,H3K27ac_Enh;E129,H3K27ac_Enh;E017,H3K9ac_Pro;E020,H3K9ac_Pro;E023,H3K9ac_Pro;E025,H3K9ac_Pro;E026,H3K9ac_Pro;E049,H3K9ac_Pro;E062,H3K9ac_Pro;E063,H3K9ac_Pro;E066,H3K9ac_Pro;E067,H3K9ac_Pro;E068,H3K9ac_Pro;E074,H3K9ac_Pro;E083,H3K9ac_Pro;E088,H3K9ac_Pro;E101,H3K9ac_Pro;E108,H3K9ac_Pro;E023,H3K4me3_Pro;E025,H3K4me3_Pro;E026,H3K4me3_Pro;E067,H3K4me3_Pro;E071,H3K4me3_Pro;E088,H3K4me3_Pro | E006;E097 | . | yes | . | Pou1f1_1;Pou3f2_1;TFIIA | GALK2 | intronic |
| 15 | rs11635005 | rs34606419 | 0.9 | G | T | 0.17 | E023,6_EnhG;E025,6_EnhG;E026,6_EnhG;E076,7_Enh;E088,7_Enh;E092,7_Enh | E023,11_TxEnh3;E025,10_TxEnh5;E026,11_TxEnh3;E049,11_TxEnh3 | E012,H3K27ac_Enh;E015,H3K27ac_Enh;E026,H3K27ac_Enh;E049,H3K27ac_Enh;E055,H3K27ac_Enh;E068,H3K27ac_Enh;E076,H3K27ac_Enh;E078,H3K27ac_Enh;E103,H3K27ac_Enh;E129,H3K27ac_Enh;E017,H3K4me1_Enh;E023,H3K4me1_Enh;E025,H3K4me1_Enh;E026,H3K4me1_Enh;E049,H3K4me1_Enh;E061,H3K4me1_Enh;E071,H3K4me1_Enh;E076,H3K4me1_Enh;E078,H3K4me1_Enh;E086,H3K4me1_Enh;E087,H3K4me1_Enh;E088,H3K4me1_Enh;E092,H3K4me1_Enh;E101,H3K4me1_Enh;E103,H3K4me1_Enh;E105,H3K4me1_Enh;E109,H3K4me1_Enh;E111,H3K4me1_Enh;E121,H3K4me1_Enh;E128,H3K4me1_Enh;E129,H3K4me1_Enh;E023,H3K4me3_Pro;E025,H3K4me3_Pro;E026,H3K4me3_Pro;E067,H3K4me3_Pro;E023,H3K9ac_Pro;E025,H3K9ac_Pro;E027,H3K9ac_Pro;E063,H3K9ac_Pro;E067,H3K9ac_Pro;E068,H3K9ac_Pro |  | . | yes | . | Mef2_disc1;Mef2_known1 | GALK2 | intronic |
| 15 | rs11635005 | rs17396119 | 0.94 | C | A | 0.15 | E025,7_Enh;E026,7_Enh;E049,7_Enh;E061,7_Enh;E121,7_Enh | E023,15_EnhAF;E025,13_EnhA1;E026,13_EnhA1;E049,15_EnhAF;E052,15_EnhAF;E120,15_EnhAF;E121,15_EnhAF;E122,18_EnhAc;E125,15_EnhAF;E126,15_EnhAF;E129,15_EnhAF | E006,H3K27ac_Enh;E015,H3K27ac_Enh;E016,H3K27ac_Enh;E017,H3K27ac_Enh;E020,H3K27ac_Enh;E026,H3K27ac_Enh;E037,H3K27ac_Enh;E049,H3K27ac_Enh;E055,H3K27ac_Enh;E056,H3K27ac_Enh;E061,H3K27ac_Enh;E078,H3K27ac_Enh;E080,H3K27ac_Enh;E120,H3K27ac_Enh;E121,H3K27ac_Enh;E124,H3K27ac_Enh;E128,H3K27ac_Enh;E129,H3K27ac_Enh;E017,H3K4me1_Enh;E020,H3K4me1_Enh;E023,H3K4me1_Enh;E025,H3K4me1_Enh;E026,H3K4me1_Enh;E049,H3K4me1_Enh;E055,H3K4me1_Enh;E061,H3K4me1_Enh;E068,H3K4me1_Enh;E071,H3K4me1_Enh;E076,H3K4me1_Enh;E078,H3K4me1_Enh;E087,H3K4me1_Enh;E088,H3K4me1_Enh;E092,H3K4me1_Enh;E103,H3K4me1_Enh;E109,H3K4me1_Enh;E120,H3K4me1_Enh;E121,H3K4me1_Enh;E123,H3K4me1_Enh;E125,H3K4me1_Enh;E126,H3K4me1_Enh;E128,H3K4me1_Enh;E129,H3K4me1_Enh;E023,H3K4me3_Pro;E025,H3K4me3_Pro;E026,H3K4me3_Pro;E121,H3K4me3_Pro;E023,H3K9ac_Pro;E025,H3K9ac_Pro;E027,H3K9ac_Pro;E049,H3K9ac_Pro;E068,H3K9ac_Pro;E072,H3K9ac_Pro;E120,H3K9ac_Pro;E121,H3K9ac_Pro | E059 | . | yes | . | DMRT2;GR_known1;HNF1_4;Hmbox1;PLZF;RORalpha1_2 | GALK2 | intronic |
| 15 | rs11635005 | rs73392277 | 0.94 | C | T | 0.17 | E017,7_Enh;E025,7_Enh;E026,7_Enh;E126,6_EnhG;E128,7_Enh;E129,7_Enh | E017,18_EnhAc;E023,15_EnhAF;E025,14_EnhA2;E026,15_EnhAF;E049,15_EnhAF;E052,15_EnhAF;E120,15_EnhAF;E121,15_EnhAF;E122,18_EnhAc;E125,15_EnhAF;E126,15_EnhAF;E128,11_TxEnh3;E129,14_EnhA2 | E006,H3K27ac_Enh;E015,H3K27ac_Enh;E017,H3K27ac_Enh;E026,H3K27ac_Enh;E037,H3K27ac_Enh;E049,H3K27ac_Enh;E056,H3K27ac_Enh;E061,H3K27ac_Enh;E108,H3K27ac_Enh;E120,H3K27ac_Enh;E121,H3K27ac_Enh;E125,H3K27ac_Enh;E126,H3K27ac_Enh;E128,H3K27ac_Enh;E129,H3K27ac_Enh;E017,H3K4me1_Enh;E023,H3K4me1_Enh;E025,H3K4me1_Enh;E026,H3K4me1_Enh;E061,H3K4me1_Enh;E076,H3K4me1_Enh;E080,H3K4me1_Enh;E083,H3K4me1_Enh;E100,H3K4me1_Enh;E104,H3K4me1_Enh;E120,H3K4me1_Enh;E121,H3K4me1_Enh;E126,H3K4me1_Enh;E128,H3K4me1_Enh;E129,H3K4me1_Enh;E017,H3K9ac_Pro;E023,H3K9ac_Pro;E025,H3K9ac_Pro;E068,H3K9ac_Pro;E121,H3K9ac_Pro;E023,H3K4me3_Pro;E025,H3K4me3_Pro;E026,H3K4me3_Pro;E076,H3K4me3_Pro;E083,H3K4me3_Pro;E121,H3K4me3_Pro |  | . | yes | . | AIRE_1;Elf3;Mef2_disc2;PLZF;PU.1_known3;p300_disc10 | GALK2 | intronic |
| 15 | rs11635005 | rs73392288 | 0.94 | G | C | 0.17 |  |  | E015,H3K27ac_Enh;E020,H3K27ac_Enh;E088,H3K4me1_Enh;E088,H3K4me3_Pro |  | . | yes | . | BATF_disc2;Hltf;Pdx1_1;Pou2f2_known3 | GALK2 | intronic |
| 15 | rs11635005 | rs7165168 | 0.94 | T | C | 0.17 |  |  | E027,H3K9ac_Pro |  | . | yes | . | Foxo_1;Foxo_2;Foxo_3;Foxo_4;RFX5_known3 | GALK2 | intronic |
| 15 | rs11635005 | rs79311354 | 0.94 | G | C | 0.17 | E026,7_Enh;E049,7_Enh;E087,7_Enh;E088,7_Enh;E108,1_TssA;E117,7_Enh;E129,7_Enh | E023,17_EnhW2;E026,17_EnhW2;E049,17_EnhW2;E052,17_EnhW2;E086,19_DNase;E088,22_PromP;E108,16_EnhW1;E114,19_DNase;E121,17_EnhW2;E129,17_EnhW2 | E002,H3K4me3_Pro;E028,H3K4me3_Pro;E100,H3K4me3_Pro;E108,H3K4me3_Pro;E012,H3K27ac_Enh;E078,H3K27ac_Enh;E089,H3K27ac_Enh;E090,H3K27ac_Enh;E108,H3K27ac_Enh;E017,H3K4me1_Enh;E026,H3K4me1_Enh;E042,H3K4me1_Enh;E049,H3K4me1_Enh;E052,H3K4me1_Enh;E071,H3K4me1_Enh;E076,H3K4me1_Enh;E078,H3K4me1_Enh;E087,H3K4me1_Enh;E088,H3K4me1_Enh;E102,H3K4me1_Enh;E108,H3K4me1_Enh;E117,H3K4me1_Enh;E120,H3K4me1_Enh;E121,H3K4me1_Enh;E129,H3K4me1_Enh;E027,H3K9ac_Pro;E088,H3K9ac_Pro;E108,H3K9ac_Pro | E017;E088;E089;E117;E120 | HeLa-S3,CEBPB,Stanford,None | yes | . | AIRE_2 | GALK2 | intronic |
| 15 | rs11635005 | rs17396536 | 0.94 | C | T | 0.15 | E017,7_Enh;E087,7_Enh;E100,7_Enh;E117,7_Enh |  | E002,H3K4me3_Pro;E100,H3K4me3_Pro;E108,H3K4me3_Pro;E012,H3K27ac_Enh;E017,H3K27ac_Enh;E089,H3K27ac_Enh;E090,H3K27ac_Enh;E108,H3K27ac_Enh;E017,H3K4me1_Enh;E026,H3K4me1_Enh;E076,H3K4me1_Enh;E087,H3K4me1_Enh;E088,H3K4me1_Enh;E100,H3K4me1_Enh;E102,H3K4me1_Enh;E108,H3K4me1_Enh;E117,H3K4me1_Enh;E027,H3K9ac_Pro;E047,H3K9ac_Pro;E088,H3K9ac_Pro |  | . | yes | . | NERF1a | GALK2 | intronic |
| 15 | rs11635005 | rs7167474 | 0.94 | C | T | 0.17 |  |  | E001,H3K4me3_Pro;E002,H3K4me3_Pro;E011,H3K4me3_Pro;E019,H3K4me3_Pro;E022,H3K4me3_Pro;E028,H3K4me1_Enh;E098,H3K4me1_Enh;E100,H3K4me1_Enh;E105,H3K4me1_Enh;E056,H3K27ac_Enh |  | . | yes | . | AFP1;Mef2_known4;Pax-4_4 | GALK2 | intronic |
| 15 | rs11635005 | rs78881953 | 0.96 | G | A | 0.15 | E017,7_Enh;E023,7_Enh;E026,7_Enh;E087,7_Enh | E023,17_EnhW2;E025,17_EnhW2 | E017,H3K27ac_Enh;E102,H3K27ac_Enh;E124,H3K27ac_Enh;E017,H3K4me1_Enh;E020,H3K4me1_Enh;E023,H3K4me1_Enh;E024,H3K4me1_Enh;E025,H3K4me1_Enh;E026,H3K4me1_Enh;E027,H3K4me1_Enh;E049,H3K4me1_Enh;E061,H3K4me1_Enh;E068,H3K4me1_Enh;E076,H3K4me1_Enh;E087,H3K4me1_Enh;E088,H3K4me1_Enh;E092,H3K4me1_Enh;E095,H3K4me1_Enh;E097,H3K4me1_Enh;E104,H3K4me1_Enh;E105,H3K4me1_Enh;E107,H3K4me1_Enh;E108,H3K4me1_Enh;E109,H3K4me1_Enh;E124,H3K4me1_Enh;E128,H3K4me1_Enh;E129,H3K4me1_Enh;E023,H3K9ac_Pro;E026,H3K4me3_Pro |  | . | yes | . | ELF1_known1;Ik-1_2;PTF1-beta;PU.1_known3;STAT_known9;UF1H3BETA;p300_known1 | GALK2 | intronic |
| 15 | rs11635005 | rs17396747 | 0.94 | G | A | 0.17 | E017,7_Enh;E023,7_Enh;E026,7_Enh | E025,17_EnhW2 | E017,H3K27ac_Enh;E049,H3K27ac_Enh;E102,H3K27ac_Enh;E108,H3K27ac_Enh;E124,H3K27ac_Enh;E017,H3K4me1_Enh;E020,H3K4me1_Enh;E023,H3K4me1_Enh;E024,H3K4me1_Enh;E025,H3K4me1_Enh;E026,H3K4me1_Enh;E027,H3K4me1_Enh;E049,H3K4me1_Enh;E061,H3K4me1_Enh;E068,H3K4me1_Enh;E076,H3K4me1_Enh;E087,H3K4me1_Enh;E088,H3K4me1_Enh;E092,H3K4me1_Enh;E095,H3K4me1_Enh;E097,H3K4me1_Enh;E104,H3K4me1_Enh;E105,H3K4me1_Enh;E107,H3K4me1_Enh;E108,H3K4me1_Enh;E109,H3K4me1_Enh;E124,H3K4me1_Enh;E128,H3K4me1_Enh;E023,H3K9ac_Pro;E026,H3K4me3_Pro |  | . | yes | . | INSM1;TR4_disc2 | GALK2 | intronic |
| 15 | rs11635005 | rs75612464 | 0.94 | T | A | 0.17 | E004,7_Enh;E011,7_Enh;E013,7_Enh;E017,7_Enh;E019,7_Enh;E023,2_TssAFlnk;E025,7_Enh;E026,2_TssAFlnk;E027,7_Enh;E049,2_TssAFlnk;E052,7_Enh;E061,7_Enh;E076,7_Enh;E107,7_Enh;E120,7_Enh;E126,7_Enh;E129,2_TssAFlnk | E004,17_EnhW2;E012,17_EnhW2;E013,18_EnhAc;E017,15_EnhAF;E019,17_EnhW2;E023,13_EnhA1;E025,13_EnhA1;E026,13_EnhA1;E028,17_EnhW2;E049,13_EnhA1;E052,14_EnhA2;E055,22_PromP;E061,22_PromP;E063,17_EnhW2;E076,22_PromP;E078,16_EnhW1;E083,17_EnhW2;E109,22_PromP;E114,18_EnhAc;E120,15_EnhAF;E121,14_EnhA2;E122,18_EnhAc;E125,14_EnhA2;E126,15_EnhAF;E128,15_EnhAF;E129,13_EnhA1 | E001,H3K4me1_Enh;E002,H3K4me1_Enh;E003,H3K4me1_Enh;E004,H3K4me1_Enh;E008,H3K4me1_Enh;E011,H3K4me1_Enh;E013,H3K4me1_Enh;E014,H3K4me1_Enh;E016,H3K4me1_Enh;E017,H3K4me1_Enh;E018,H3K4me1_Enh;E019,H3K4me1_Enh;E020,H3K4me1_Enh;E021,H3K4me1_Enh;E023,H3K4me1_Enh;E024,H3K4me1_Enh;E025,H3K4me1_Enh;E026,H3K4me1_Enh;E027,H3K4me1_Enh;E028,H3K4me1_Enh;E049,H3K4me1_Enh;E052,H3K4me1_Enh;E055,H3K4me1_Enh;E059,H3K4me1_Enh;E061,H3K4me1_Enh;E063,H3K4me1_Enh;E065,H3K4me1_Enh;E067,H3K4me1_Enh;E069,H3K4me1_Enh;E071,H3K4me1_Enh;E074,H3K4me1_Enh;E076,H3K4me1_Enh;E087,H3K4me1_Enh;E088,H3K4me1_Enh;E092,H3K4me1_Enh;E095,H3K4me1_Enh;E097,H3K4me1_Enh;E100,H3K4me1_Enh;E104,H3K4me1_Enh;E105,H3K4me1_Enh;E107,H3K4me1_Enh;E108,H3K4me1_Enh;E109,H3K4me1_Enh;E120,H3K4me1_Enh;E121,H3K4me1_Enh;E125,H3K4me1_Enh;E126,H3K4me1_Enh;E128,H3K4me1_Enh;E129,H3K4me1_Enh;E003,H3K27ac_Enh;E004,H3K27ac_Enh;E005,H3K27ac_Enh;E012,H3K27ac_Enh;E016,H3K27ac_Enh;E017,H3K27ac_Enh;E026,H3K27ac_Enh;E049,H3K27ac_Enh;E061,H3K27ac_Enh;E063,H3K27ac_Enh;E065,H3K27ac_Enh;E079,H3K27ac_Enh;E095,H3K27ac_Enh;E101,H3K27ac_Enh;E102,H3K27ac_Enh;E104,H3K27ac_Enh;E108,H3K27ac_Enh;E109,H3K27ac_Enh;E120,H3K27ac_Enh;E121,H3K27ac_Enh;E125,H3K27ac_Enh;E128,H3K27ac_Enh;E129,H3K27ac_Enh;E017,H3K4me3_Pro;E023,H3K4me3_Pro;E025,H3K4me3_Pro;E026,H3K4me3_Pro;E049,H3K4me3_Pro;E129,H3K4me3_Pro;E017,H3K9ac_Pro;E023,H3K9ac_Pro;E025,H3K9ac_Pro;E026,H3K9ac_Pro;E049,H3K9ac_Pro;E102,H3K9ac_Pro | E017;E059;E117;E119;E120;E128 | . | yes | . | Dmbx1;Gsc | GALK2 | intronic |
| 15 | rs11635005 | rs73394324 | 0.94 | C | T | 0.17 | E026,7_Enh;E030,7_Enh | E025,17_EnhW2 | E003,H3K9ac_Pro;E004,H3K27ac_Enh;E007,H3K27ac_Enh;E017,H3K27ac_Enh;E049,H3K27ac_Enh;E097,H3K27ac_Enh;E015,H3K4me1_Enh;E023,H3K4me1_Enh;E026,H3K4me1_Enh;E027,H3K4me1_Enh;E030,H3K4me1_Enh;E049,H3K4me1_Enh;E055,H3K4me1_Enh;E061,H3K4me1_Enh;E067,H3K4me1_Enh;E069,H3K4me1_Enh;E071,H3K4me1_Enh;E072,H3K4me1_Enh;E076,H3K4me1_Enh;E083,H3K4me1_Enh;E087,H3K4me1_Enh;E095,H3K4me1_Enh;E108,H3K4me1_Enh |  | . | yes | . | CTCF_disc4;Ets_disc5;Mrg_2;Sin3Ak-20_disc4;Tgif1_1;Tgif1_2 | GALK2 | intronic |
| 15 | rs11635005 | rs111805023 | 0.92 | C | T | 0.17 |  |  | E091,H3K27ac_Enh;E095,H3K4me1_Enh |  | . | yes | . | ZBTB33_disc4 | GALK2 | intronic |
| 15 | rs11635005 | rs73394331 | 0.94 | G | A | 0.17 | E061,7_Enh |  | E002,H3K4me1_Enh;E061,H3K4me1_Enh;E095,H3K4me1_Enh |  | . | yes | . | EBF_disc2;NRSF_known1 | GALK2 | intronic |
| 15 | rs11635005 | rs73394333 | 0.94 | C | T | 0.17 |  |  | E002,H3K4me1_Enh;E011,H3K4me1_Enh;E014,H3K4me1_Enh;E020,H3K4me1_Enh;E024,H3K4me1_Enh;E026,H3K4me1_Enh;E030,H3K4me1_Enh;E068,H3K4me1_Enh;E129,H3K4me1_Enh;E108,H3K27ac_Enh;E121,H3K9ac_Pro;E124,H3K9ac_Pro |  | . | yes | . | Barx2;Dbx1;Dbx2;HNF1_6;HNF1_7;Hlxb9;Hmbox1;Hoxb8;Hoxc6;Hoxd8;Msx-1_2;Ncx_2;Nkx6-1_2 | GALK2 | intronic |
| 15 | rs11635005 | rs73394339 | 0.94 | A | G | 0.17 | E017,7_Enh;E030,7_Enh;E120,7_Enh;E129,7_Enh |  | E002,H3K4me1_Enh;E014,H3K4me1_Enh;E017,H3K4me1_Enh;E020,H3K4me1_Enh;E024,H3K4me1_Enh;E026,H3K4me1_Enh;E030,H3K4me1_Enh;E049,H3K4me1_Enh;E068,H3K4me1_Enh;E069,H3K4me1_Enh;E077,H3K4me1_Enh;E090,H3K4me1_Enh;E097,H3K4me1_Enh;E100,H3K4me1_Enh;E107,H3K4me1_Enh;E108,H3K4me1_Enh;E120,H3K4me1_Enh;E121,H3K4me1_Enh;E129,H3K4me1_Enh;E075,H3K27ac_Enh;E084,H3K27ac_Enh;E089,H3K27ac_Enh;E090,H3K27ac_Enh;E100,H3K27ac_Enh;E101,H3K27ac_Enh;E108,H3K27ac_Enh;E109,H3K27ac_Enh;E121,H3K27ac_Enh;E121,H3K9ac_Pro;E124,H3K9ac_Pro | E050;E120 | . | yes | . | AP-1_disc8;Maf_known3;Maf_known4 | GALK2 | intronic |
| 15 | rs11635005 | rs17397211 | 0.94 | A | G | 0.17 | E017,7_Enh;E020,7_Enh;E030,7_Enh;E107,7_Enh;E120,7_Enh;E121,7_Enh;E129,7_Enh | E023,17_EnhW2;E026,17_EnhW2;E052,19_DNase;E068,17_EnhW2;E107,18_EnhAc;E108,17_EnhW2;E120,17_EnhW2;E121,17_EnhW2;E126,17_EnhW2;E129,17_EnhW2 | E002,H3K4me1_Enh;E014,H3K4me1_Enh;E017,H3K4me1_Enh;E020,H3K4me1_Enh;E024,H3K4me1_Enh;E026,H3K4me1_Enh;E030,H3K4me1_Enh;E049,H3K4me1_Enh;E068,H3K4me1_Enh;E069,H3K4me1_Enh;E077,H3K4me1_Enh;E090,H3K4me1_Enh;E095,H3K4me1_Enh;E097,H3K4me1_Enh;E100,H3K4me1_Enh;E107,H3K4me1_Enh;E108,H3K4me1_Enh;E120,H3K4me1_Enh;E121,H3K4me1_Enh;E129,H3K4me1_Enh;E026,H3K4me3_Pro;E075,H3K27ac_Enh;E084,H3K27ac_Enh;E089,H3K27ac_Enh;E090,H3K27ac_Enh;E100,H3K27ac_Enh;E101,H3K27ac_Enh;E108,H3K27ac_Enh;E109,H3K27ac_Enh;E121,H3K27ac_Enh;E121,H3K9ac_Pro;E124,H3K9ac_Pro | E006;E080;E084;E100;E120;E126 | . | yes | . | CEBPB_known2 | GALK2 | intronic |
| 15 | rs11635005 | rs76069611 | 0.94 | G | A | 0.16 | E026,7_Enh | E026,17_EnhW2;E049,18_EnhAc | E017,H3K4me1_Enh;E023,H3K4me1_Enh;E026,H3K4me1_Enh;E027,H3K4me1_Enh;E049,H3K4me1_Enh;E052,H3K4me1_Enh;E065,H3K4me1_Enh;E100,H3K4me1_Enh;E108,H3K4me1_Enh;E121,H3K4me1_Enh;E129,H3K4me1_Enh;E023,H3K4me3_Pro;E026,H3K4me3_Pro;E100,H3K4me3_Pro;E026,H3K27ac_Enh;E075,H3K27ac_Enh;E089,H3K27ac_Enh;E101,H3K27ac_Enh |  | . | yes | . | DMRT7;Nanog_disc1;Nanog_disc4;Pou2f2_disc1;Pou2f2_known7;Pou2f2_known8;Pou3f3;Pou5f1_disc1;Pou5f1_disc2;Pou5f1_known1;TATA_disc9 | GALK2 | intronic |
| 15 | rs11635005 | rs10519221 | 0.94 | A | G | 0.17 |  |  | E011,H3K4me1_Enh;E013,H3K4me1_Enh;E015,H3K4me1_Enh;E016,H3K4me1_Enh;E025,H3K4me1_Enh;E026,H3K4me1_Enh;E049,H3K4me1_Enh;E061,H3K4me1_Enh;E065,H3K4me1_Enh;E088,H3K4me1_Enh;E097,H3K4me1_Enh;E106,H3K4me1_Enh;E109,H3K4me1_Enh;E117,H3K4me1_Enh;E122,H3K4me1_Enh;E129,H3K4me1_Enh;E011,H3K4me3_Pro;E026,H3K4me3_Pro;E017,H3K27ac_Enh;E026,H3K27ac_Enh;E044,H3K27ac_Enh;E049,H3K27ac_Enh;E078,H3K27ac_Enh;E102,H3K27ac_Enh;E124,H3K27ac_Enh;E017,H3K9ac_Pro |  | . | yes | . | Fox;Irx;Nkx2_5;Sox_7 | GALK2 | intronic |
| 15 | rs11635005 | rs78050772 | 0.96 | G | A | 0.15 | E011,7_Enh;E026,6_EnhG;E049,6_EnhG;E061,6_EnhG;E088,6_EnhG;E117,7_Enh;E122,7_Enh | E026,11_TxEnh3;E049,11_TxEnh3;E122,11_TxEnh3;E129,11_TxEnh3 | E011,H3K4me1_Enh;E013,H3K4me1_Enh;E015,H3K4me1_Enh;E016,H3K4me1_Enh;E025,H3K4me1_Enh;E026,H3K4me1_Enh;E049,H3K4me1_Enh;E061,H3K4me1_Enh;E065,H3K4me1_Enh;E088,H3K4me1_Enh;E097,H3K4me1_Enh;E106,H3K4me1_Enh;E109,H3K4me1_Enh;E117,H3K4me1_Enh;E122,H3K4me1_Enh;E129,H3K4me1_Enh;E011,H3K4me3_Pro;E026,H3K4me3_Pro;E017,H3K27ac_Enh;E026,H3K27ac_Enh;E044,H3K27ac_Enh;E049,H3K27ac_Enh;E078,H3K27ac_Enh;E102,H3K27ac_Enh;E124,H3K27ac_Enh;E017,H3K9ac_Pro |  | . | yes | . | Fox;Nkx2_5;Sox_7 | GALK2 | intronic |
| 15 | rs11635005 | rs57749497 | 0.96 | C | G | 0.17 |  |  | E056,H3K4me1_Enh;E058,H3K27ac_Enh |  | . | yes | . | Zfp410 | GALK2 | intronic |
| 15 | rs11635005 | rs73394355 | 0.96 | A | G | 0.17 |  |  | E010,H3K4me1_Enh;E027,H3K4me1_Enh;E056,H3K4me1_Enh;E110,H3K4me1_Enh;E117,H3K4me1_Enh;E015,H3K27ac_Enh;E102,H3K9ac_Pro |  | . | yes | . | Mtf1_1;Mtf1_2 | GALK2 | intronic |
| 15 | rs11635005 | rs73394359 | 0.96 | G | A | 0.17 | E005,7_Enh;E106,6_EnhG |  | E015,H3K27ac_Enh;E025,H3K4me1_Enh;E026,H3K4me1_Enh;E027,H3K4me1_Enh;E049,H3K4me1_Enh;E055,H3K4me1_Enh;E056,H3K4me1_Enh;E095,H3K4me1_Enh;E109,H3K4me1_Enh;E110,H3K4me1_Enh;E117,H3K4me1_Enh;E127,H3K4me1_Enh;E047,H3K9ac_Pro;E121,H3K4me3_Pro |  | . | yes | . | FAC1 | GALK2 | intronic |
| 15 | rs11635005 | rs17476361 | 0.98 | A | G | 0.15 | E123,7_Enh |  | E023,H3K4me1_Enh;E025,H3K4me1_Enh;E027,H3K4me1_Enh;E055,H3K4me1_Enh;E056,H3K4me1_Enh;E095,H3K4me1_Enh;E109,H3K4me1_Enh;E110,H3K4me1_Enh;E123,H3K4me1_Enh;E129,H3K4me1_Enh;E058,H3K27ac_Enh |  | . | yes | . | CIZ;Gfi1_1;Gfi1_2;Hdx;Hoxa4;Pax-4_2;Pax-4_5;p300_disc5 | GALK2 | intronic |
| 15 | rs11635005 | rs11635005 | 1 | C | T | 0.17 | E107,7_Enh;E108,7_Enh |  | E026,H3K4me1_Enh;E027,H3K4me1_Enh;E059,H3K4me1_Enh;E061,H3K4me1_Enh;E083,H3K4me1_Enh;E095,H3K4me1_Enh;E100,H3K4me1_Enh;E104,H3K4me1_Enh;E105,H3K4me1_Enh;E107,H3K4me1_Enh;E108,H3K4me1_Enh;E109,H3K4me1_Enh;E121,H3K4me1_Enh;E027,H3K9ac_Pro;E061,H3K27ac_Enh;E107,H3K4me3_Pro | E128 | . | yes | yes | Irf_known9;Pou5f1_known2;STAT_disc3;Zfp187 | GALK2 | intronic |
| 15 | rs11635005 | rs112074680 | 1 | C | T | 0.17 | E061,7_Enh;E108,7_Enh |  | E026,H3K4me1_Enh;E059,H3K4me1_Enh;E061,H3K4me1_Enh;E083,H3K4me1_Enh;E095,H3K4me1_Enh;E100,H3K4me1_Enh;E104,H3K4me1_Enh;E105,H3K4me1_Enh;E107,H3K4me1_Enh;E108,H3K4me1_Enh;E121,H3K4me1_Enh;E061,H3K27ac_Enh;E107,H3K4me3_Pro |  | . | yes | . | Mef2_disc1 | GALK2 | intronic |
| 15 | rs11635005 | rs11630318 | 1 | A | T | 0.17 | E055,7_Enh |  | E007,H3K9ac_Pro;E110,H3K9ac_Pro;E015,H3K27ac_Enh;E016,H3K27ac_Enh;E017,H3K27ac_Enh;E020,H3K27ac_Enh;E111,H3K27ac_Enh;E017,H3K4me1_Enh;E025,H3K4me1_Enh;E026,H3K4me1_Enh;E027,H3K4me1_Enh;E028,H3K4me1_Enh;E049,H3K4me1_Enh;E055,H3K4me1_Enh;etc | E055;E100 | . | yes | . | Cphx;HP1-site-factor;OTX;Pitx2;TEF;p300_disc6 | GALK2 | intronic |
| 15 | rs11635005 | rs11632038 | 1 | A | G | 0.15 | E017,7_Enh;E023,7_Enh;E025,7_Enh;E026,7_Enh;E028,7_Enh;E052,7_Enh;E055,7_Enh;E057,7_Enh;E058,7_Enh;E076,7_Enh;E090,7_Enh;E097,7_Enh;E108,7_Enh;E111,7_Enh;E119,7_Enh;E120,7_Enh;E121,7_Enh;E126,7_Enh;E129,7_Enh | E006,17_EnhW2;E017,17_EnhW2;E023,17_EnhW2;E025,14_EnhA2;E026,17_EnhW2;E028,17_EnhW2;E049,17_EnhW2;E052,17_EnhW2;E055,14_EnhA2;E056,17_EnhW2;E057,17_EnhW2;E063,17_EnhW2;E076,17_EnhW2;E078,17_EnhW2;E089,17_EnhW2;E103,17_EnhW2;E104,17_EnhW2;E106,17_EnhW2;E107,17_EnhW2;E108,16_EnhW1;E111,17_EnhW2;E117,17_EnhW2;E119,17_EnhW2;E120,15_EnhAF;E121,17_EnhW2;E125,17_EnhW2;E126,15_EnhAF;E128,17_EnhW2;E129,14_EnhA2 | E001,H3K4me1_Enh;E002,H3K4me1_Enh;E013,H3K4me1_Enh;E015,H3K4me1_Enh;E016,H3K4me1_Enh;E017,H3K4me1_Enh;E019,H3K4me1_Enh;E020,H3K4me1_Enh;E023,H3K4me1_Enh;E024,H3K4me1_Enh;E025,H3K4me1_Enh;E026,H3K4me1_Enh;E028,H3K4me1_Enh;E052,H3K4me1_Enh;E053,H3K4me1_Enh;E055,H3K4me1_Enh;E056,H3K4me1_Enh;E057,H3K4me1_Enh;E058,H3K4me1_Enh;E063,H3K4me1_Enh;E065,H3K4me1_Enh;E067,H3K4me1_Enh;E068,H3K4me1_Enh;E071,H3K4me1_Enh;E073,H3K4me1_Enh;E074,H3K4me1_Enh;E076,H3K4me1_Enh;E078,H3K4me1_Enh;E079,H3K4me1_Enh;E088,H3K4me1_Enh;E089,H3K4me1_Enh;E090,H3K4me1_Enh;E092,H3K4me1_Enh;E094,H3K4me1_Enh;E095,H3K4me1_Enh;E097,H3K4me1_Enh;E100,H3K4me1_Enh;E103,H3K4me1_Enh;E104,H3K4me1_Enh;E105,H3K4me1_Enh;E106,H3K4me1_Enh;E107,H3K4me1_Enh;E108,H3K4me1_Enh;E109,H3K4me1_Enh;E110,H3K4me1_Enh;E111,H3K4me1_Enh;E117,H3K4me1_Enh;E119,H3K4me1_Enh;E120,H3K4me1_Enh;E121,H3K4me1_Enh;E125,H3K4me1_Enh;E126,H3K4me1_Enh;E128,H3K4me1_Enh;E129,H3K4me1_Enh;E015,H3K27ac_Enh;E017,H3K27ac_Enh;E020,H3K27ac_Enh;E021,H3K27ac_Enh;E026,H3K27ac_Enh;E090,H3K27ac_Enh;E092,H3K27ac_Enh;E098,H3K27ac_Enh;E102,H3K27ac_Enh;E111,H3K27ac_Enh;E121,H3K27ac_Enh;E129,H3K27ac_Enh;E015,H3K9ac_Pro;etc |  | . | yes | . | Barhl1;Barx2;En-1_3;Gbx1;Hlx1;Hmbox1;Hoxa4;Lhx4;Msx2;Nkx2_8;Nobox_1;Prrx2_1 | GALK2 | intronic |
| 15 | rs11635005 | rs11634636 | 1 | G | A | 0.15 | E001,7_Enh;E003,7_Enh;E004,7_Enh;E006,7_Enh;E008,7_Enh;E009,7_Enh;E010,7_Enh;E011,7_Enh;E013,7_Enh;E014,7_Enh;E015,7_Enh;E016,7_Enh;E018,7_Enh;E019,7_Enh;E020,7_Enh;E023,7_Enh;E024,7_Enh;E025,7_Enh;E053,7_Enh;E054,7_Enh;E063,7_Enh;E067,7_Enh;E074,7_Enh;E076,7_Enh;etc | E001,13_EnhA1;E002,15_EnhAF;E003,14_EnhA2;E004,14_EnhA2;E005,17_EnhW2;E007,17_EnhW2;E008,15_EnhAF;E009,15_EnhAF;E010,15_EnhAF;E011,15_EnhAF;E012,15_EnhAF;E013,15_EnhAF;E014,14_EnhA2;E015,14_EnhA2;E016,15_EnhAF;E018,15_EnhAF;E019,13_EnhA1;E020,14_EnhA2;E021,15_EnhAF;E022,17_EnhW2;E023,17_EnhW2;E024,15_EnhAF;E027,17_EnhW2;E053,19_DNase;E054,16_EnhW1;E063,17_EnhW2;etc | E001,H3K4me1_Enh;E002,H3K4me1_Enh;E003,H3K4me1_Enh;E004,H3K4me1_Enh;E006,H3K4me1_Enh;E008,H3K4me1_Enh;E009,H3K4me1_Enh;E010,H3K4me1_Enh;E011,H3K4me1_Enh;E012,H3K4me1_Enh;E013,H3K4me1_Enh;E014,H3K4me1_Enh;E015,H3K4me1_Enh;E016,H3K4me1_Enh;E017,H3K4me1_Enh;E018,H3K4me1_Enh;E019,H3K4me1_Enh;E020,H3K4me1_Enh;E023,H3K4me1_Enh;E024,H3K4me1_Enh;E025,H3K4me1_Enh;E053,H3K4me1_Enh;E054,H3K4me1_Enh;E055,H3K4me1_Enh;E056,H3K4me1_Enh;E063,H3K4me1_Enh;E067,H3K4me1_Enh;E068,H3K4me1_Enh;E071,H3K4me1_Enh;E073,H3K4me1_Enh;E074,H3K4me1_Enh;E076,H3K4me1_Enh;E078,H3K4me1_Enh;E079,H3K4me1_Enh;E085,H3K4me1_Enh;E088,H3K4me1_Enh;E089,H3K4me1_Enh;E090,H3K4me1_Enh;E092,H3K4me1_Enh;E095,H3K4me1_Enh;E097,H3K4me1_Enh;E100,H3K4me1_Enh;E103,H3K4me1_Enh;E104,H3K4me1_Enh;E105,H3K4me1_Enh;E107,H3K4me1_Enh;E108,H3K4me1_Enh;E109,H3K4me1_Enh;E111,H3K4me1_Enh;E125,H3K4me1_Enh;E129,H3K4me1_Enh;E001,H3K4me3_Pro;E019,H3K4me3_Pro;E020,H3K4me3_Pro;E076,H3K4me3_Pro;E111,H3K4me3_Pro;E003,H3K27ac_Enh;E004,H3K27ac_Enh;E008,H3K27ac_Enh;E014,H3K27ac_Enh;E015,H3K27ac_Enh;E016,H3K27ac_Enh;etc | E004;E089;E090;E091;E092 | . | yes | . | . | GALK2 | intronic |
| 15 | rs11635005 | rs35692325 | 1 | T | C | 0.17 | E001,7_Enh;E002,7_Enh;E003,7_Enh;E004,7_Enh;E014,7_Enh;E015,7_Enh;E016,7_Enh;E018,7_Enh;E019,7_Enh;E020,7_Enh;E092,7_Enh | E001,15_EnhAF;E002,17_EnhW2;E003,17_EnhW2;E004,17_EnhW2;E008,17_EnhW2;E011,17_EnhW2;E012,17_EnhW2;E014,15_EnhAF;E015,17_EnhW2;E016,15_EnhAF;E018,17_EnhW2;E019,15_EnhAF;E020,16_EnhW1;E021,17_EnhW2;E024,17_EnhW2 | E001,H3K4me1_Enh;E002,H3K4me1_Enh;E003,H3K4me1_Enh;E004,H3K4me1_Enh;E011,H3K4me1_Enh;E012,H3K4me1_Enh;E013,H3K4me1_Enh;E014,H3K4me1_Enh;E015,H3K4me1_Enh;E016,H3K4me1_Enh;E018,H3K4me1_Enh;E019,H3K4me1_Enh;E020,H3K4me1_Enh;E023,H3K4me1_Enh;E024,H3K4me1_Enh;E025,H3K4me1_Enh;E055,H3K4me1_Enh;E076,H3K4me1_Enh;E092,H3K4me1_Enh;E095,H3K4me1_Enh;E107,H3K4me1_Enh;E001,H3K4me3_Pro;E019,H3K4me3_Pro;E020,H3K4me3_Pro;E076,H3K4me3_Pro;E003,H3K27ac_Enh;E004,H3K27ac_Enh;E014,H3K27ac_Enh;E015,H3K27ac_Enh;E016,H3K27ac_Enh;E019,H3K27ac_Enh;E020,H3K27ac_Enh;E021,H3K27ac_Enh;E015,H3K9ac_Pro;E020,H3K9ac_Pro | E089;E090 | . | yes | . | Irf_disc3;Zfp410 | GALK2 | intronic |
| 15 | rs11635005 | rs7162633 | 0.98 | G | A | 0.17 |  |  | E001,H3K4me1_Enh;E005,H3K4me1_Enh;E009,H3K4me1_Enh;E014,H3K4me1_Enh;E015,H3K4me1_Enh;E019,H3K4me1_Enh;E092,H3K4me1_Enh;E096,H3K4me1_Enh;E097,H3K4me1_Enh;E109,H3K4me1_Enh;E003,H3K27ac_Enh;E019,H3K27ac_Enh;E022,H3K27ac_Enh;E014,H3K9ac_Pro |  | . | yes | . | LUN-1;Mxi1_disc1;Pitx2 | GALK2 | intronic |
| 15 | rs11635005 | rs74339964 | 0.98 | T | C | 0.15 | E001,7_Enh;E002,7_Enh;E014,7_Enh;E015,7_Enh;E016,7_Enh;E018,7_Enh;E019,7_Enh;E020,7_Enh | E001,15_EnhAF;E002,17_EnhW2;E003,17_EnhW2;E004,17_EnhW2;E008,17_EnhW2;E009,17_EnhW2;E011,17_EnhW2;E012,17_EnhW2;E014,15_EnhAF;E015,17_EnhW2;E016,15_EnhAF;E018,15_EnhAF;E019,15_EnhAF;E020,15_EnhAF;E022,17_EnhW2;E024,17_EnhW2 | E001,H3K4me1_Enh;E002,H3K4me1_Enh;E003,H3K4me1_Enh;E004,H3K4me1_Enh;E013,H3K4me1_Enh;E014,H3K4me1_Enh;E015,H3K4me1_Enh;E016,H3K4me1_Enh;E018,H3K4me1_Enh;E019,H3K4me1_Enh;E020,H3K4me1_Enh;E021,H3K4me1_Enh;E024,H3K4me1_Enh;E025,H3K4me1_Enh;E055,H3K4me1_Enh;E061,H3K4me1_Enh;E068,H3K4me1_Enh;E128,H3K4me1_Enh;E003,H3K27ac_Enh;E004,H3K27ac_Enh;E014,H3K27ac_Enh;E015,H3K27ac_Enh;E016,H3K27ac_Enh;E019,H3K27ac_Enh;E020,H3K27ac_Enh;E021,H3K27ac_Enh;E014,H3K4me3_Pro;E018,H3K4me3_Pro;E019,H3K4me3_Pro;E020,H3K4me3_Pro;E014,H3K9ac_Pro;E016,H3K9ac_Pro;E019,H3K9ac_Pro;E020,H3K9ac_Pro |  | . | yes | . | Gm397 | C15orf33 | intronic |
| 15 | rs11635005 | rs200924420 | 0.96 | TTGGCCTGCCTTGC | T | 0.17 |  |  |  |  | . | yes | . | NF-kappaB_known4;PPAR_1;PU.1_known3 | C15orf33 | intronic |
| 15 | rs11635005 | rs111600039 | 0.96 | C | T | 0.17 | E001,7_Enh;E015,7_Enh;E018,7_Enh | E001,17_EnhW2;E002,17_EnhW2;E008,17_EnhW2;E015,17_EnhW2;E016,17_EnhW2;E018,17_EnhW2;E020,17_EnhW2 | E001,H3K4me1_Enh;E014,H3K4me1_Enh;E015,H3K4me1_Enh;E016,H3K4me1_Enh;E022,H3K4me1_Enh;E034,H3K4me1_Enh;E113,H3K4me1_Enh;E001,H3K9ac_Pro;E008,H3K9ac_Pro;E015,H3K27ac_Enh;E016,H3K27ac_Enh;E056,H3K27ac_Enh |  | . | yes | . | Hsf_known1 | C15orf33 | intronic |
| 15 | rs11635005 | rs111307556 | 0.96 | T | C | 0.17 | E001,7_Enh;E015,7_Enh | E001,16_EnhW1;E003,17_EnhW2;E004,17_EnhW2;E008,17_EnhW2;E015,17_EnhW2;E016,17_EnhW2;E018,17_EnhW2;E019,17_EnhW2 | E001,H3K4me1_Enh;E005,H3K4me1_Enh;E014,H3K4me1_Enh;E015,H3K4me1_Enh;E016,H3K4me1_Enh;E018,H3K4me1_Enh;E022,H3K4me1_Enh;E024,H3K4me1_Enh;E034,H3K4me1_Enh;E113,H3K4me1_Enh;E001,H3K4me3_Pro;E022,H3K4me3_Pro;E001,H3K9ac_Pro;E008,H3K9ac_Pro;E015,H3K27ac_Enh;E016,H3K27ac_Enh;E022,H3K27ac_Enh;E056,H3K27ac_Enh |  | . | yes | . | Foxa_disc3;GR_disc5;HEY1_disc2;NRSF_disc8;Nanog_disc3;Sin3Ak-20_disc7 | C15orf33 | intronic |
| 15 | rs11635005 | rs73396235 | 0.96 | A | T | 0.17 |  |  | E011,H3K4me1_Enh;E012,H3K4me1_Enh;E014,H3K4me1_Enh;E015,H3K4me1_Enh;E016,H3K4me1_Enh;E019,H3K4me1_Enh;E061,H3K4me1_Enh;E012,H3K27ac_Enh;E020,H3K27ac_Enh |  | . | yes | . | . | C15orf33 | intronic |
| 15 | rs11635005 | rs73396238 | 0.96 | G | A | 0.17 |  |  | E011,H3K4me1_Enh;E012,H3K4me1_Enh;E015,H3K4me1_Enh;E016,H3K4me1_Enh;E019,H3K4me1_Enh;E061,H3K4me1_Enh;E012,H3K27ac_Enh;E020,H3K27ac_Enh |  | . | yes | . | FEV;HMG-IY_1 | C15orf33 | intronic |
| 15 | rs11635005 | rs57252806 | 0.96 | G | A | 0.17 |  | E023,19_DNase;E025,19_DNase;E026,19_DNase;E055,19_DNase;E083,19_DNase;E128,19_DNase;E129,19_DNase | E002,H3K4me3_Pro;E055,H3K27ac_Enh;E056,H3K27ac_Enh;E113,H3K4me1_Enh | E126 | . | yes | . | DBP;Foxa_disc1;Foxa_known1;Foxa_known3;Foxa_known4;Foxj2_1;HDAC2_disc2;HNF4_disc4;TCF12_disc2;Zfp410;p300_disc3 | C15orf33 | intronic |
| 15 | rs11635005 | rs139884542 | 0.98 | G | A | 0.15 |  |  | E002,H3K4me3_Pro |  | . | yes | . | E2A_3;E2A_4;NF-kappaB_disc3;SREBP_known4;ZEB1_known4 | C15orf33 | intronic |
| 15 | rs11635005 | rs143359226 | 0.96 | G | C | 0.17 |  |  |  |  | . | yes | . | EWSR1-FLI1 | C15orf33 | intronic |
| 15 | rs11635005 | rs11633981 | 0.94 | A | G | 0.17 |  |  |  |  | . | yes | . | . | C15orf33 | intronic |
| 15 | rs11635005 | rs139995312 | 0.94 | GGTC | G | 0.17 |  |  |  |  | . | . | . | Evi-1_4;Nkx2_3;TCF4_disc2 | C15orf33 | intronic |
| 15 | rs11635005 | rs112854186 | 0.96 | C | G | 0.15 |  |  | E012,H3K27ac_Enh |  | . | yes | . | SIX5_known2;SZF1-1;Spz1_2 | C15orf33 | intronic |
| 15 | rs11635005 | rs11633282 | 0.94 | C | T | 0.16 | E001,7_Enh;E002,7_Enh;E004,7_Enh;E009,7_Enh;E010,7_Enh;E011,7_Enh;E014,7_Enh;E015,7_Enh;E016,7_Enh;E018,7_Enh;E019,7_Enh;E020,7_Enh;E024,7_Enh | E001,16_EnhW1;E002,17_EnhW2;E003,17_EnhW2;E004,16_EnhW1;E007,19_DNase;E008,17_EnhW2;E009,16_EnhW1;E010,17_EnhW2;E011,16_EnhW1;E012,17_EnhW2;E013,17_EnhW2;E014,16_EnhW1;E015,16_EnhW1;E016,16_EnhW1;E018,16_EnhW1;E019,16_EnhW1;E020,16_EnhW1;E021,17_EnhW2;E022,17_EnhW2;E024,17_EnhW2;E083,17_EnhW2 | E001,H3K4me1_Enh;E002,H3K4me1_Enh;E003,H3K4me1_Enh;E004,H3K4me1_Enh;E007,H3K4me1_Enh;E008,H3K4me1_Enh;E009,H3K4me1_Enh;E010,H3K4me1_Enh;E011,H3K4me1_Enh;E012,H3K4me1_Enh;E014,H3K4me1_Enh;E015,H3K4me1_Enh;E016,H3K4me1_Enh;E018,H3K4me1_Enh;E019,H3K4me1_Enh;E020,H3K4me1_Enh;E021,H3K4me1_Enh;E024,H3K4me1_Enh;E117,H3K4me1_Enh;E008,H3K27ac_Enh;E014,H3K27ac_Enh;E011,H3K4me3_Pro;E014,H3K4me3_Pro;E019,H3K4me3_Pro;E020,H3K4me3_Pro |  | . | yes | . | Arid3a_2;CHX10;Esx1;Hoxa10;Hoxd10;Pou3f2_4;RORalpha1_1 | C15orf33 | intronic |
| 15 | rs11635005 | rs11638606 | 0.94 | T | C | 0.16 | E001,7_Enh;E002,7_Enh;E004,7_Enh;E009,7_Enh;E010,7_Enh;E011,7_Enh;E014,7_Enh;E015,7_Enh;E016,7_Enh;E018,7_Enh;E019,7_Enh;E020,7_Enh;E024,7_Enh | E001,16_EnhW1;E002,17_EnhW2;E003,17_EnhW2;E004,16_EnhW1;E007,19_DNase;E008,17_EnhW2;E009,16_EnhW1;E010,17_EnhW2;E011,16_EnhW1;E012,17_EnhW2;E013,17_EnhW2;E014,16_EnhW1;E015,16_EnhW1;E016,16_EnhW1;E018,16_EnhW1;E019,16_EnhW1;E020,16_EnhW1;E021,17_EnhW2;E022,17_EnhW2;E024,17_EnhW2;E083,17_EnhW2 | E001,H3K4me1_Enh;E002,H3K4me1_Enh;E003,H3K4me1_Enh;E004,H3K4me1_Enh;E007,H3K4me1_Enh;E008,H3K4me1_Enh;E009,H3K4me1_Enh;E010,H3K4me1_Enh;E011,H3K4me1_Enh;E012,H3K4me1_Enh;E014,H3K4me1_Enh;E015,H3K4me1_Enh;E016,H3K4me1_Enh;E018,H3K4me1_Enh;E019,H3K4me1_Enh;E020,H3K4me1_Enh;E021,H3K4me1_Enh;E024,H3K4me1_Enh;E117,H3K4me1_Enh;E008,H3K27ac_Enh;E014,H3K27ac_Enh;E011,H3K4me3_Pro;E014,H3K4me3_Pro;E019,H3K4me3_Pro;E020,H3K4me3_Pro | E003;E004;E008;E021;E022 | H1-hESC,POU5F1,HudsonAlpha,None;H1-hESC,USF1,HudsonAlpha,None | yes | . | ERalpha-a_disc1;ERalpha-a_known2;ERalpha-a_known4;HNF4_known4;RAR;VDR_1 | C15orf33 | intronic |
| 15 | rs11635005 | rs35533804 | 0.94 | ATC | A | 0.16 |  |  | E001,H3K4me1_Enh;E002,H3K4me1_Enh;E011,H3K4me1_Enh;E014,H3K4me1_Enh;E015,H3K4me1_Enh;E016,H3K4me1_Enh;E018,H3K4me1_Enh;E020,H3K4me1_Enh;E011,H3K4me3_Pro;E014,H3K27ac_Enh;E015,H3K27ac_Enh;E047,H3K9ac_Pro |  | . | yes | . | Cdc5;Cdx2_2;Foxm1;GATA_disc4;GATA_known10;GATA_known2;Hoxa10;Hoxd10;TCF11::MafG | C15orf33 | intronic |
| 15 | rs11635005 | rs77408643 | 0.96 | C | T | 0.15 |  |  |  |  | . | yes | . | DMRT2;Sox_13;Sox_17 | C15orf33 | intronic |
| 15 | rs11635005 | rs148331731 | 0.94 | GATCAT | G | 0.16 |  |  | E055,H3K27ac_Enh |  | . | yes | . | DMRT2;Evi-1_4;Foxp1;GATA_known1;HMG-IY_2;Irf_known3;Irf_known9;Zfp105;p300_disc5 | C15orf33 | intronic |
| 15 | rs11635005 | rs73398223 | 0.92 | G | C | 0.17 |  |  | E002,H3K4me1_Enh;E055,H3K27ac_Enh |  | . | yes | . | Cdx;Foxf2;Foxl1_1;Mef2_disc1;TATA_known3 | C15orf33 | intronic |
| 15 | rs11635005 | rs60594237 | 0.94 | C | T | 0.15 | E021,7_Enh;E036,2_TssAFlnk | E036,22_PromP | E001,H3K4me1_Enh;E002,H3K4me1_Enh;E006,H3K4me1_Enh;E011,H3K4me1_Enh;E012,H3K4me1_Enh;E017,H3K4me1_Enh;E018,H3K4me1_Enh;E021,H3K4me1_Enh;E024,H3K4me1_Enh;E036,H3K4me1_Enh;E005,H3K9ac_Pro;E055,H3K27ac_Enh;E098,H3K4me3_Pro;E113,H3K4me3_Pro |  | . | yes | . | AP-2_disc2;AP-2_known6;AP-2_known7;NF-kappaB_disc2 | C15orf33 | intronic |
| 15 | rs11635005 | rs11632575 | 0.94 | T | G | 0.16 | E002,7_Enh;E003,7_Enh;E015,7_Enh;E016,7_Enh;E019,7_Enh;E021,7_Enh | E001,17_EnhW2;E002,17_EnhW2;E004,17_EnhW2;E008,17_EnhW2;E014,17_EnhW2;E018,17_EnhW2;E020,17_EnhW2;E022,17_EnhW2 | E001,H3K4me1_Enh;E002,H3K4me1_Enh;E003,H3K4me1_Enh;E004,H3K4me1_Enh;E005,H3K4me1_Enh;E008,H3K4me1_Enh;E011,H3K4me1_Enh;E012,H3K4me1_Enh;E014,H3K4me1_Enh;E015,H3K4me1_Enh;E016,H3K4me1_Enh;E018,H3K4me1_Enh;E019,H3K4me1_Enh;E020,H3K4me1_Enh;E021,H3K4me1_Enh;E022,H3K4me1_Enh;E024,H3K4me1_Enh;E036,H3K4me1_Enh;E093,H3K4me1_Enh;E004,H3K27ac_Enh;E005,H3K27ac_Enh;E014,H3K27ac_Enh;E020,H3K27ac_Enh;E055,H3K27ac_Enh;E020,H3K4me3_Pro;E027,H3K9ac_Pro |  | . | yes | . | Nkx2_11;Nkx2_3;Nkx2_4;Nkx2_7;Nkx3_3;RORalpha1_2 | C15orf33 | intronic |
| 15 | rs11635005 | rs77296528 | 0.96 | G | A | 0.15 |  |  | E001,H3K4me1_Enh;E002,H3K4me1_Enh;E011,H3K4me1_Enh;E012,H3K4me1_Enh;E015,H3K4me1_Enh;E016,H3K4me1_Enh;E018,H3K4me1_Enh;E019,H3K4me1_Enh;E020,H3K4me1_Enh;E024,H3K4me1_Enh;E117,H3K4me1_Enh;E020,H3K27ac_Enh |  | . | yes | . | Arid3a_2;Cdx2_1;Hoxa10;Nkx2_10;Pax-2_2;Pdx1_2;Pou2f2_known10;TATA_disc9 | C15orf33 | intronic |
| 15 | rs11635005 | rs59112977 | 0.94 | C | T | 0.16 | E018,7_Enh |  | E001,H3K4me1_Enh;E002,H3K4me1_Enh;E010,H3K4me1_Enh;E011,H3K4me1_Enh;E012,H3K4me1_Enh;E015,H3K4me1_Enh;E016,H3K4me1_Enh;E018,H3K4me1_Enh;E019,H3K4me1_Enh;E020,H3K4me1_Enh;E024,H3K4me1_Enh;E117,H3K4me1_Enh;E020,H3K27ac_Enh;E027,H3K9ac_Pro |  | . | yes | . | Foxa_known3;Foxd1_2;Foxj1_2;Foxj2_2;Foxk1;HDAC2_disc2;HNF1_5;HNF1_6;HNF1_7;Nkx6-1_1;Pou4f3;p300_disc3 | C15orf33 | intronic |
| 15 | rs11635005 | rs8027094 | 0.94 | C | G | 0.16 |  |  |  |  | . | yes | . | Arid3a_2;Barhl1;CHX10;Esx1;Foxa_known4;Hoxa10;Hoxa3_2;Hoxa5_3;Hoxa9;Hoxb13;Hoxb9;Hoxd10;Lhx3_2;Lhx4;Mef2_known5;Msx2;Pax7;Pou2f2_known4;Pou3f2_4;Pou6f1_2;Prrx2_1;RORalpha1_2 | C15orf33 | intronic |
| 15 | rs11635005 | rs8030457 | 0.96 | G | A | 0.16 |  |  | E001,H3K4me1_Enh;E009,H3K4me1_Enh;E010,H3K4me1_Enh;E012,H3K4me1_Enh;E015,H3K4me1_Enh;E016,H3K4me1_Enh;E018,H3K4me1_Enh;E019,H3K4me1_Enh;E020,H3K4me1_Enh;E021,H3K4me1_Enh;E024,H3K4me1_Enh;E019,H3K4me3_Pro |  | . | yes | . | Barx1;Barx2;Dbx2;En-1_3;Gbx1;Hlxb9;Hoxa5_3;Hoxb3;Hoxb7;Msx-1_2;Msx2;Pax-6_3;Pax7;Pou2f2_known4;Pou3f2_4;Pou3f4;Pou6f1_2;Prrx2_1;RXRA_known4;Vax2 | C15orf33 | intronic |
| 15 | rs11635005 | rs17477722 | 0.96 | G | A | 0.16 |  | E001,17_EnhW2;E015,16_EnhW1;E019,17_EnhW2;E020,16_EnhW1 | E001,H3K4me1_Enh;E008,H3K4me1_Enh;E012,H3K4me1_Enh;E014,H3K4me1_Enh;E015,H3K4me1_Enh;E016,H3K4me1_Enh;E018,H3K4me1_Enh;E019,H3K4me1_Enh;E020,H3K4me1_Enh;E024,H3K4me1_Enh;E011,H3K9ac_Pro;E016,H3K9ac_Pro;E019,H3K9ac_Pro;E015,H3K4me3_Pro;E016,H3K4me3_Pro;E019,H3K4me3_Pro;E020,H3K4me3_Pro |  | . | yes | yes | Bsx;Dlx2;Foxc1_1;Prrx2_1 | C15orf33 | intronic |
| 15 | rs11635005 | rs73398243 | 0.96 | G | A | 0.16 |  | E001,17_EnhW2;E015,16_EnhW1;E019,17_EnhW2;E020,16_EnhW1 | E001,H3K4me3_Pro;E015,H3K4me3_Pro;E016,H3K4me3_Pro;E019,H3K4me3_Pro;E020,H3K4me3_Pro;E024,H3K4me3_Pro;E011,H3K9ac_Pro;E016,H3K9ac_Pro;E019,H3K9ac_Pro;E012,H3K4me1_Enh;E014,H3K4me1_Enh;E015,H3K4me1_Enh;E016,H3K4me1_Enh;E018,H3K4me1_Enh;E019,H3K4me1_Enh;E020,H3K4me1_Enh;E024,H3K4me1_Enh |  | . | yes | . | Maf_known2;NF-kappaB_disc2 | C15orf33 | intronic |
| 15 | rs11635005 | rs79365293 | 0.96 | G | A | 0.15 |  |  | E008,H3K27ac_Enh |  | . | yes | . | BATF_disc3 | C15orf33 | intronic |
| 15 | rs11635005 | rs113885253 | 0.96 | A | T | 0.16 |  |  | E008,H3K27ac_Enh;E012,H3K27ac_Enh | E123 | . | yes | . | . | C15orf33 | intronic |
| 15 | rs11635005 | rs73398250 | 0.96 | G | T | 0.16 |  |  |  |  | . | yes | . | AIRE_1;Foxf1;Foxi1;Foxl1_1;Foxo_1;Foxo_2;Foxo_3;Foxq1 | C15orf33 | intronic |
| 15 | rs11635005 | rs1023683 | 0.9 | T | A | 0.17 |  |  | E012,H3K27ac_Enh;E017,H3K27ac_Enh;E128,H3K27ac_Enh;E129,H3K27ac_Enh;E017,H3K4me1_Enh;E023,H3K4me1_Enh;E025,H3K4me1_Enh;E026,H3K4me1_Enh;E028,H3K4me1_Enh;E030,H3K4me1_Enh;E049,H3K4me1_Enh;E052,H3K4me1_Enh;E055,H3K4me1_Enh;E056,H3K4me1_Enh;E058,H3K4me1_Enh;E061,H3K4me1_Enh;E103,H3K4me1_Enh;E117,H3K4me1_Enh;E119,H3K4me1_Enh;E122,H3K4me1_Enh;E125,H3K4me1_Enh;E126,H3K4me1_Enh;E128,H3K4me1_Enh;E129,H3K4me1_Enh;E024,H3K4me3_Pro;E025,H3K4me3_Pro;E088,H3K4me3_Pro;E119,H3K9ac_Pro | E006;E017;E055;E117 | HeLa-S3,CEBPB,Stanford,None | yes | . | Foxa_disc2;SRF_known3;SRF_known5 | C15orf33 | intronic |
| 15 | rs11635005 | rs11639423 | 0.9 | T | A | 0.17 | E023,7_Enh;E025,7_Enh;E026,7_Enh;E049,7_Enh;E052,2_TssAFlnk;E117,7_Enh;E119,7_Enh;E122,7_Enh;E125,7_Enh;E126,7_Enh;E127,7_Enh;E128,7_Enh;E129,7_Enh | E006,17_EnhW2;E017,17_EnhW2;E023,16_EnhW1;E025,16_EnhW1;E026,17_EnhW2;E028,19_DNase;E049,17_EnhW2;E052,16_EnhW1;E055,19_DNase;E056,18_EnhAc;E059,19_DNase;E088,19_DNase;E114,19_DNase;E117,18_EnhAc;E119,17_EnhW2;E121,17_EnhW2;E122,17_EnhW2;E125,17_EnhW2;E126,16_EnhW1;E127,19_DNase;E128,17_EnhW2;E129,16_EnhW1 | E012,H3K27ac_Enh;E017,H3K27ac_Enh;E128,H3K27ac_Enh;E129,H3K27ac_Enh;E023,H3K4me1_Enh;E025,H3K4me1_Enh;E026,H3K4me1_Enh;E028,H3K4me1_Enh;E049,H3K4me1_Enh;E052,H3K4me1_Enh;E055,H3K4me1_Enh;E056,H3K4me1_Enh;E058,H3K4me1_Enh;E061,H3K4me1_Enh;E092,H3K4me1_Enh;E103,H3K4me1_Enh;E110,H3K4me1_Enh;E117,H3K4me1_Enh;E119,H3K4me1_Enh;E121,H3K4me1_Enh;E122,H3K4me1_Enh;E125,H3K4me1_Enh;E126,H3K4me1_Enh;E127,H3K4me1_Enh;E128,H3K4me1_Enh;E129,H3K4me1_Enh;E024,H3K4me3_Pro;E025,H3K4me3_Pro;E052,H3K4me3_Pro;E119,H3K9ac_Pro | E006;E117;E119 | . | yes | . | HNF1_6;HNF1_7 | C15orf33 | intronic |
| 15 | rs11635005 | rs73398272 | 0.96 | G | A | 0.16 | E056,7_Enh;E092,7_Enh;E117,7_Enh;E128,7_Enh | E006,18_EnhAc;E017,17_EnhW2;E055,18_EnhAc;E056,15_EnhAF;E117,18_EnhAc;E128,15_EnhAF | E012,H3K27ac_Enh;E017,H3K27ac_Enh;E056,H3K27ac_Enh;E117,H3K27ac_Enh;E128,H3K27ac_Enh;E129,H3K27ac_Enh;E023,H3K4me1_Enh;E025,H3K4me1_Enh;E026,H3K4me1_Enh;E028,H3K4me1_Enh;E052,H3K4me1_Enh;E055,H3K4me1_Enh;E056,H3K4me1_Enh;E057,H3K4me1_Enh;E076,H3K4me1_Enh;E092,H3K4me1_Enh;E103,H3K4me1_Enh;E117,H3K4me1_Enh;E121,H3K4me1_Enh;E125,H3K4me1_Enh;E126,H3K4me1_Enh;E127,H3K4me1_Enh;E128,H3K4me1_Enh;E129,H3K4me1_Enh;E076,H3K4me3_Pro;E097,H3K4me3_Pro | E017;E117 | . | yes | . | . | C15orf33 | intronic |
| 15 | rs11635005 | rs73398276 | 0.96 | C | A | 0.16 |  |  | E017,H3K4me1_Enh;E025,H3K4me1_Enh;E062,H3K4me1_Enh;E063,H3K9ac_Pro;E097,H3K27ac_Enh;E097,H3K4me3_Pro |  | . | yes | . | CCNT2_disc1;Foxa_disc5;Foxp1;GATA_known14;HDAC2_disc6;TAL1_disc1;TCF4_known1;Zfp105;p300_disc5 | C15orf33 | intronic |
| 15 | rs11635005 | rs11635354 | 0.96 | G | C | 0.15 |  |  | E025,H3K4me1_Enh;E062,H3K4me1_Enh;E049,H3K9ac_Pro;E063,H3K9ac_Pro;E068,H3K9ac_Pro;E083,H3K9ac_Pro;E097,H3K4me3_Pro |  | . | yes | . | Barhl1;HNF4_disc4;Maf_known4 | C15orf33 | intronic |
| 15 | rs11635005 | rs11638314 | 0.96 | G | A | 0.15 | E023,7_Enh;E025,7_Enh;E026,7_Enh;E071,7_Enh;E076,7_Enh;E088,7_Enh;E103,7_Enh | E023,13_EnhA1;E025,13_EnhA1;E026,16_EnhW1;E049,16_EnhW1;E069,17_EnhW2;E071,22_PromP;E076,16_EnhW1;E078,16_EnhW1;E103,17_EnhW2;E111,16_EnhW1 | E001,H3K4me1_Enh;E011,H3K4me1_Enh;E014,H3K4me1_Enh;E016,H3K4me1_Enh;E019,H3K4me1_Enh;E020,H3K4me1_Enh;E023,H3K4me1_Enh;E025,H3K4me1_Enh;E026,H3K4me1_Enh;E027,H3K4me1_Enh;E040,H3K4me1_Enh;E042,H3K4me1_Enh;E049,H3K4me1_Enh;E066,H3K4me1_Enh;E069,H3K4me1_Enh;E071,H3K4me1_Enh;E074,H3K4me1_Enh;E076,H3K4me1_Enh;E078,H3K4me1_Enh;E083,H3K4me1_Enh;E084,H3K4me1_Enh;E085,H3K4me1_Enh;etc |  | . | yes | . | Hoxa5_1;Irf_known7;Nrf1_disc1 | C15orf33 | intronic |
| 15 | rs11635005 | rs73398284 | 0.92 | T | C | 0.17 | E014,7_Enh;E016,7_Enh;E020,7_Enh;E023,1_TssA;E025,1_TssA;E026,2_TssAFlnk;E027,7_Enh;E043,7_Enh;E049,1_TssA;E051,1_TssA;E063,1_TssA;E065,1_TssA;E066,1_TssA;E069,7_Enh;etc | E001,19_DNase;E002,19_DNase;E003,19_DNase;E004,19_DNase;E005,19_DNase;E006,19_DNase;E007,19_DNase;E008,19_DNase;E009,19_DNase;E010,19_DNase;E011,19_DNase;E012,19_DNase;E013,19_DNase;E014,19_DNase;E015,19_DNase;E016,22_PromP;E017,19_DNase;etc | E001,H3K4me1_Enh;E011,H3K4me1_Enh;E014,H3K4me1_Enh;E015,H3K4me1_Enh;E016,H3K4me1_Enh;E019,H3K4me1_Enh;E020,H3K4me1_Enh;E023,H3K4me1_Enh;E024,H3K4me1_Enh;E025,H3K4me1_Enh;E026,H3K4me1_Enh;E027,H3K4me1_Enh;E040,H3K4me1_Enh;E042,H3K4me1_Enh;E043,H3K4me1_Enh;E049,H3K4me1_Enh;E050,H3K4me1_Enh;E051,H3K4me1_Enh;E063,H3K4me1_Enh;E066,H3K4me1_Enh;E068,H3K4me1_Enh;E069,H3K4me1_Enh;E071,H3K4me1_Enh;E072,H3K4me1_Enh;E074,H3K4me1_Enh;E076,H3K4me1_Enh;E078,H3K4me1_Enh;E081,H3K4me1_Enh;E083,H3K4me1_Enh;E084,H3K4me1_Enh;E085,H3K4me1_Enh;E087,H3K4me1_Enh;E088,H3K4me1_Enh;E092,H3K4me1_Enh;E102,H3K4me1_Enh;E103,H3K4me1_Enh;E107,H3K4me1_Enh;E108,H3K4me1_Enh;E111,H3K4me1_Enh;E122,H3K4me1_Enh;E125,H3K4me1_Enh;etc | E003;E004;E006;E007;E008;E028;E029;E032;E033;E034;E046;E050;E051;E055;E056;E059;E082;E083;E086;E088;E089;E090;E091;E092;E093;E094;E097;E098;E118;E119;E121;E122;E123;E124;E126 | AG09309,CTCF,UW,None;AG09319,CTCF,UW,None;AG10803,CTCF,UW,None;BJ,CTCF,UW,None;GM06990,CTCF,UW,None;GM12864,CTCF,  etc | yes | . | . | C15orf33 | intronic |
| 15 | rs11635005 | rs11635308 | 0.98 | G | T | 0.15 | E017,3_TxFlnk;E023,3_TxFlnk;E025,3_TxFlnk;E026,3_TxFlnk;E049,2_TssAFlnk;E051,7_Enh;E063,1_TssA;E076,1_TssA;E078,1_TssA;E083,7_Enh;E086,1_TssA;E090,1_TssA;E092,1_TssA;E097,1_TssA;E101,1_TssA;E103,1_TssA;E104,1_TssA;E105,1_TssA;E111,1_TssA;E126,1_TssA;E128,1_TssA;E129,1_TssA | E006,22_PromP;E011,22_PromP;E013,22_PromP;E017,4_PromD2;E023,9_TxReg;E025,4_PromD2;E026,4_PromD2;E027,22_PromP;E028,22_PromP;E049,4_PromD2;E051,22_PromP;E052,22_PromP;E055,22_PromP;E056,22_PromP;E063,4_PromD2;E065,22_PromP;E066,22_PromP;E071,22_PromP;E075,22_PromP;E076,4_PromD2;E078,4_PromD2;E080,22_PromP;E083,4_PromD2;E086,22_PromP;E087,22_PromP;E088,4_PromD2;E089,22_PromP;E090,22_PromP;E091,22_PromP; etc | E014,H3K4me1_Enh;E020,H3K4me1_Enh;E023,H3K4me1_Enh;E026,H3K4me1_Enh;E049,H3K4me1_Enh;E050,H3K4me1_Enh;E083,H3K4me1_Enh;E088,H3K4me1_Enh;E103,H3K4me1_Enh;E014,H3K9ac_Pro;E017,H3K9ac_Pro;E023,H3K9ac_Pro;E025,H3K9ac_Pro;E026,H3K9ac_Pro;E027,H3K9ac_Pro;E049,H3K9ac_Pro;E063,H3K9ac_Pro;E076,H3K9ac_Pro;E083,H3K9ac_Pro;E086,H3K9ac_Pro;E088,H3K9ac_Pro;E101,H3K9ac_Pro;E102,H3K9ac_Pro;E107,H3K9ac_Pro;E108,H3K9ac_Pro;E111,H3K9ac_Pro;E120,H3K9ac_Pro;E126,H3K9ac_Pro;E128,H3K9ac_Pro;E017,H3K27ac_Enh;E026,H3K27ac_Enh;E049,H3K27ac_Enh;E063,H3K27ac_Enh;E075,H3K27ac_Enh;E076,H3K27ac_Enh;E078,H3K27ac_Enh;E102,H3K27ac_Enh;E103,H3K27ac_Enh;E111,H3K27ac_Enh;E126,H3K27ac_Enh;E128,H3K27ac_Enh;E129,H3K27ac_Enh;E017,H3K4me3_Pro;E023,H3K4me3_Pro;E025,H3K4me3_Pro;E026,H3K4me3_Pro;E049,H3K4me3_Pro;E051,H3K4me3_Pro;E063,H3K4me3_Pro;E075,H3K4me3_Pro;E076,H3K4me3_Pro;E078,H3K4me3_Pro;E083,H3K4me3_Pro;E084,H3K4me3_Pro;E085,H3K4me3_Pro;E086,H3K4me3_Pro;E088,H3K4me3_Pro;E089,H3K4me3_Pro;E090,H3K4me3_Pro;E092,H3K4me3_Pro;E097,H3K4me3_Pro;E101,H3K4me3_Pro;E102,H3K4me3_Pro;E103,H3K4me3_Pro;E104,H3K4me3_Pro;E105,H3K4me3_Pro;E108,H3K4me3_Pro;E111,H3K4me3_Pro;E121,H3K4me3_Pro;E126,H3K4me3_Pro;E128,H3K4me3_Pro;E129,H3K4me3_Pro |  | . | yes | . | HP1-site-factor;Pou2f2_known2;Pou2f2_known8;Pou3f1 | FGF7 | intronic |
| 15 | rs11635005 | rs61259878 | 0.98 | C | G | 0.16 | E023,3_TxFlnk;E025,3_TxFlnk;E026,3_TxFlnk;E049,7_Enh;E129,7_Enh | E023,9_TxReg;E025,4_PromD2;E026,9_TxReg;E049,4_PromD2;E126,22_PromP;E129,12_TxEnhW | E017,H3K27ac_Enh;E026,H3K27ac_Enh;E038,H3K27ac_Enh;E049,H3K27ac_Enh;E063,H3K27ac_Enh;E076,H3K27ac_Enh;E078,H3K27ac_Enh;E103,H3K27ac_Enh;E111,H3K27ac_Enh;E017,H3K4me1_Enh;E023,H3K4me1_Enh;E025,H3K4me1_Enh;E026,H3K4me1_Enh;E049,H3K4me1_Enh;E076,H3K4me1_Enh;E078,H3K4me1_Enh;E083,H3K4me1_Enh;E092,H3K4me1_Enh;E103,H3K4me1_Enh;E105,H3K4me1_Enh;E126,H3K4me1_Enh;E129,H3K4me1_Enh;E017,H3K4me3_Pro;E023,H3K4me3_Pro;E025,H3K4me3_Pro;E026,H3K4me3_Pro;E049,H3K4me3_Pro;E063,H3K4me3_Pro;E076,H3K4me3_Pro;E078,H3K4me3_Pro;E103,H3K4me3_Pro;E111,H3K4me3_Pro;E129,H3K4me3_Pro;E017,H3K9ac_Pro;E023,H3K9ac_Pro;E025,H3K9ac_Pro;E026,H3K9ac_Pro;E049,H3K9ac_Pro |  | . | yes | . | HNF1_7;Nkx2_1;PLZF | FGF7 | intronic |
| 15 | rs11635005 | rs78323502 | 0.98 | G | T | 0.15 | E017,7_Enh;E023,2_TssAFlnk;E025,3_TxFlnk;E026,3_TxFlnk;E049,2_TssAFlnk;E050,7_Enh;E051,7_Enh;E052,7_Enh;E076,7_Enh;E083,7_Enh;E086,7_Enh;E088,7_Enh;E092,7_Enh;E103,7_Enh;E111,1_TssA;E120,7_Enh;E121,7_Enh;E126,7_Enh;E128,7_Enh;E129,2_TssAFlnk | E006,17_EnhW2;E017,10_TxEnh5;E023,9_TxReg;E025,9_TxReg;E026,9_TxReg;E049,9_TxReg;E052,14_EnhA2;E063,14_EnhA2;E074,19_DNase;E076,14_EnhA2;E078,10_TxEnh5;E081,17_EnhW2;E083,10_TxEnh5;E086,17_EnhW2;E087,18_EnhAc;E088,14_EnhA2;E089,17_EnhW2;E090,17_EnhW2;E092,18_EnhAc;E097,16_EnhW1;E103,13_EnhA1;E111,16_EnhW1;E120,15_EnhAF;E121,15_EnhAF;E122,17_EnhW2;E125,15_EnhAF;E126,14_EnhA2;E128,14_EnhA2;E129,9_TxReg | E006,H3K27ac_Enh;E017,H3K27ac_Enh;E026,H3K27ac_Enh;E038,H3K27ac_Enh;E049,H3K27ac_Enh;E050,H3K27ac_Enh;E063,H3K27ac_Enh;E075,H3K27ac_Enh;E076,H3K27ac_Enh;E078,H3K27ac_Enh;E089,H3K27ac_Enh;E090,H3K27ac_Enh;E095,H3K27ac_Enh;E097,H3K27ac_Enh;E101,H3K27ac_Enh;E103,H3K27ac_Enh;E111,H3K27ac_Enh;E120,H3K27ac_Enh;E125,H3K27ac_Enh;E129,H3K27ac_Enh;E017,H3K4me1_Enh;E023,H3K4me1_Enh;E025,H3K4me1_Enh;E026,H3K4me1_Enh;E035,H3K4me1_Enh;E036,H3K4me1_Enh;E049,H3K4me1_Enh;E050,H3K4me1_Enh;E051,H3K4me1_Enh;E052,H3K4me1_Enh;E063,H3K4me1_Enh;E071,H3K4me1_Enh;E074,H3K4me1_Enh;E076,H3K4me1_Enh;E077,H3K4me1_Enh;E078,H3K4me1_Enh;E083,H3K4me1_Enh;E086,H3K4me1_Enh;E088,H3K4me1_Enh;E092,H3K4me1_Enh;E097,H3K4me1_Enh;E102,H3K4me1_Enh;E103,H3K4me1_Enh;E111,H3K4me1_Enh;E117,H3K4me1_Enh;E120,H3K4me1_Enh;E121,H3K4me1_Enh;E125,H3K4me1_Enh;E126,H3K4me1_Enh;E128,H3K4me1_Enh;E129,H3K4me1_Enh;E017,H3K9ac_Pro;E023,H3K9ac_Pro;E025,H3K9ac_Pro;E026,H3K9ac_Pro;E047,H3K9ac_Pro;E049,H3K9ac_Pro;E083,H3K9ac_Pro;E088,H3K9ac_Pro;E120,H3K9ac_Pro;E023,H3K4me3_Pro;E025,H3K4me3_Pro;E026,H3K4me3_Pro;E049,H3K4me3_Pro;E063,H3K4me3_Pro;E076,H3K4me3_Pro;E078,H3K4me3_Pro;E083,H3K4me3_Pro;E102,H3K4me3_Pro;E103,H3K4me3_Pro;E111,H3K4me3_Pro;E129,H3K4me3_Pro | E017;E089;E090;E117 | . | yes | . | HDAC2_disc3;Smad_2 | FGF7 | intronic |
| 15 | rs11635005 | rs200066768 | 0.98 | A | AT | 0.15 | E017,7_Enh;E023,2_TssAFlnk;E025,3_TxFlnk;E026,3_TxFlnk;E049,2_TssAFlnk;E050,7_Enh;E051,7_Enh;E052,7_Enh;E076,7_Enh;E083,7_Enh;E086,7_Enh;E088,7_Enh;E092,7_Enh;E103,7_Enh;E111,1_TssA;E120,7_Enh;E121,7_Enh;E126,7_Enh;E128,7_Enh;E129,2_TssAFlnk | E006,17_EnhW2;E017,10_TxEnh5;E023,9_TxReg;E025,9_TxReg;E026,9_TxReg;E049,9_TxReg;E052,14_EnhA2;E063,14_EnhA2;E074,19_DNase;E076,14_EnhA2;E078,10_TxEnh5;E081,17_EnhW2;E083,10_TxEnh5;E086,17_EnhW2;E087,18_EnhAc;E088,14_EnhA2;E089,17_EnhW2;E090,17_EnhW2;E092,18_EnhAc;E097,16_EnhW1;E103,13_EnhA1;E111,16_EnhW1;E120,15_EnhAF;E121,15_EnhAF;E122,17_EnhW2; etc | E006,H3K27ac_Enh;E017,H3K27ac_Enh;E026,H3K27ac_Enh;E038,H3K27ac_Enh;E049,H3K27ac_Enh;E050,H3K27ac_Enh;E063,H3K27ac_Enh;E075,H3K27ac_Enh;E076,H3K27ac_Enh;E078,H3K27ac_Enh;E089,H3K27ac_Enh;E090,H3K27ac_Enh;E095,H3K27ac_Enh;E097,H3K27ac_Enh;E101,H3K27ac_Enh;E103,H3K27ac_Enh;E111,H3K27ac_Enh;E120,H3K27ac_Enh;E125,H3K27ac_Enh;E129,H3K27ac_Enh;E017,H3K4me1_Enh;E023,H3K4me1_Enh;E025,H3K4me1_Enh;E026,H3K4me1_Enh;E035,H3K4me1_Enh;E036,H3K4me1_Enh;E049,H3K4me1_Enh;E050,H3K4me1_Enh;E051,H3K4me1_Enh;E052,H3K4me1_Enh;E063,H3K4me1_Enh;E071,H3K4me1_Enh;E074,H3K4me1_Enh;E076,H3K4me1_Enh;E077,H3K4me1_Enh;E078,H3K4me1_Enh;E083,H3K4me1_Enh;E086,H3K4me1_Enh;E088,H3K4me1_Enh;E092,H3K4me1_Enh;E097,H3K4me1_Enh;E102,H3K4me1_Enh;E103,H3K4me1_Enh;E111,H3K4me1_Enh;E117,H3K4me1_Enh;E120,H3K4me1_Enh;E121,H3K4me1_Enh;E125,H3K4me1_Enh;E126,H3K4me1_Enh;E128,H3K4me1_Enh;E129,H3K4me1_Enh;E017,H3K9ac_Pro;E023,H3K9ac_Pro;E025,H3K9ac_Pro;E026,H3K9ac_Pro;E047,H3K9ac_Pro;E049,H3K9ac_Pro;E083,H3K9ac_Pro;E088,H3K9ac_Pro;E120,H3K9ac_Pro; etc | E017;E088;E089;E090;E117;E126 | . | yes | . | Nkx2_8;Pou3f4 | FGF7 | intronic |
| 15 | rs11635005 | rs79404431 | 0.98 | C | A | 0.15 | E017,7_Enh;E023,2_TssAFlnk;E025,3_TxFlnk;E026,3_TxFlnk;E049,2_TssAFlnk;E050,7_Enh;E051,7_Enh;E052,7_Enh;E076,7_Enh;E083,7_Enh;E086,7_Enh;E088,7_Enh;E092,7_Enh;E103,7_Enh;E111,1_TssA;E120,7_Enh;E121,7_Enh;E126,7_Enh;E128,7_Enh;E129,2_TssAFlnk | E006,17_EnhW2;E017,10_TxEnh5;E023,9_TxReg;E025,9_TxReg;E026,9_TxReg;E049,9_TxReg;E052,14_EnhA2;E063,14_EnhA2;E074,19_DNase;E076,14_EnhA2;E078,10_TxEnh5;E081,17_EnhW2;E083,10_TxEnh5;E086,17_EnhW2;E087,18_EnhAc;E088,14_EnhA2;E089,17_EnhW2;E090,17_EnhW2;E092,18_EnhAc;E097,16_EnhW1;E103,13_EnhA1;E111,16_EnhW1;E120,15_EnhAF;E121,15_EnhAF;E122,17_EnhW2;E125,15_EnhAF;E126,14_EnhA2; etc | E006,H3K27ac_Enh;E017,H3K27ac_Enh;E026,H3K27ac_Enh;E038,H3K27ac_Enh;E049,H3K27ac_Enh;E050,H3K27ac_Enh;E063,H3K27ac_Enh;E075,H3K27ac_Enh;E076,H3K27ac_Enh;E078,H3K27ac_Enh;E089,H3K27ac_Enh;E090,H3K27ac_Enh;E095,H3K27ac_Enh;E097,H3K27ac_Enh;E101,H3K27ac_Enh;E103,H3K27ac_Enh;E111,H3K27ac_Enh;E120,H3K27ac_Enh;E125,H3K27ac_Enh;E129,H3K27ac_Enh;E017,H3K4me1_Enh;E023,H3K4me1_Enh;E025,H3K4me1_Enh;E026,H3K4me1_Enh;E035,H3K4me1_Enh;E036,H3K4me1_Enh;E049,H3K4me1_Enh;E050,H3K4me1_Enh;E051,H3K4me1_Enh;E052,H3K4me1_Enh;E063,H3K4me1_Enh;E071,H3K4me1_Enh;E074,H3K4me1_Enh;E076,H3K4me1_Enh;E077,H3K4me1_Enh;E078,H3K4me1_Enh;E083,H3K4me1_Enh;E086,H3K4me1_Enh;E088,H3K4me1_Enh;E092,H3K4me1_Enh;E097,H3K4me1_Enh;E102,H3K4me1_Enh;E103,H3K4me1_Enh;E111,H3K4me1_Enh;E117,H3K4me1_Enh;E120,H3K4me1_Enh;E121,H3K4me1_Enh;E125,H3K4me1_Enh;E126,H3K4me1_Enh;E128,H3K4me1_Enh;E129,H3K4me1_Enh;E017,H3K9ac_Pro;E023,H3K9ac_Pro;E025,H3K9ac_Pro;E026,H3K9ac_Pro;E047,H3K9ac_Pro;E049,H3K9ac_Pro;E083,H3K9ac_Pro;E088,H3K9ac_Pro;E120,H3K9ac_Pro;E023,H3K4me3_Pro;E025,H3K4me3_Pro;E026,H3K4me3_Pro;E049,H3K4me3_Pro;E063,H3K4me3_Pro; etc | E088;E089;E090;E117;E126 | . | yes | . | HMG-IY_2;Nkx3_1;Pou3f4 | FGF7 | intronic |
| 15 | rs11635005 | rs17478618 | 0.98 | G | A | 0.16 | E017,6_EnhG;E023,2_TssAFlnk;E025,3_TxFlnk;E026,6_EnhG;E049,7_Enh;E076,7_Enh;E092,7_Enh;E097,7_Enh;E103,7_Enh;E111,1_TssA;E129,7_Enh | E023,9_TxReg;E025,9_TxReg;E026,10_TxEnh5;E049,4_PromD2;E052,17_EnhW2;E076,18_EnhAc;E111,16_EnhW1;E129,18_EnhAc | E003,H3K9ac_Pro;E017,H3K9ac_Pro;E023,H3K9ac_Pro;E025,H3K9ac_Pro;E049,H3K9ac_Pro;E066,H3K9ac_Pro;E017,H3K27ac_Enh;E049,H3K27ac_Enh;E063,H3K27ac_Enh;E103,H3K27ac_Enh;E111,H3K27ac_Enh;E017,H3K4me1_Enh;E023,H3K4me1_Enh;E025,H3K4me1_Enh;E026,H3K4me1_Enh;E049,H3K4me1_Enh;E076,H3K4me1_Enh;E078,H3K4me1_Enh;E087,H3K4me1_Enh;E092,H3K4me1_Enh;E097,H3K4me1_Enh;E103,H3K4me1_Enh;E105,H3K4me1_Enh;E111,H3K4me1_Enh;E129,H3K4me1_Enh;E023,H3K4me3_Pro;E025,H3K4me3_Pro;E026,H3K4me3_Pro;E076,H3K4me3_Pro;E111,H3K4me3_Pro | E088 | . | yes | . | PPAR_1 | FGF7 | intronic |
| 15 | rs11635005 | rs10519226 | 0.92 | T | C | 0.17 |  |  | E023,H3K4me1_Enh;E025,H3K4me1_Enh;E026,H3K4me1_Enh;E049,H3K4me1_Enh;E076,H3K4me1_Enh;E078,H3K4me1_Enh;E111,H3K4me1_Enh;E129,H3K4me1_Enh;E023,H3K4me3_Pro;E025,H3K4me3_Pro;E026,H3K4me3_Pro;E111,H3K4me3_Pro;E023,H3K9ac_Pro;E025,H3K9ac_Pro;E049,H3K9ac_Pro;E026,H3K27ac_Enh;E049,H3K27ac_Enh;E063,H3K27ac_Enh;E076,H3K27ac_Enh;E103,H3K27ac_Enh;E111,H3K27ac_Enh |  | . | yes | . | Hoxd10 | FGF7 | intronic |
| 15 | rs11635005 | rs17400427 | 0.92 | T | C | 0.17 | E026,6_EnhG;E083,7_Enh;E103,7_Enh | E023,10_TxEnh5;E025,12_TxEnhW | E017,H3K4me1_Enh;E023,H3K4me1_Enh;E025,H3K4me1_Enh;E026,H3K4me1_Enh;E049,H3K4me1_Enh;E051,H3K4me1_Enh;E063,H3K4me1_Enh;E076,H3K4me1_Enh;E078,H3K4me1_Enh;E083,H3K4me1_Enh;E088,H3K4me1_Enh;E092,H3K4me1_Enh;E097,H3K4me1_Enh;E103,H3K4me1_Enh;E111,H3K4me1_Enh;E129,H3K4me1_Enh;E023,H3K4me3_Pro;E026,H3K4me3_Pro;E023,H3K9ac_Pro;E025,H3K9ac_Pro;E026,H3K9ac_Pro;E049,H3K9ac_Pro;E076,H3K9ac_Pro;E026,H3K27ac_Enh;E049,H3K27ac_Enh;E063,H3K27ac_Enh;E076,H3K27ac_Enh;E103,H3K27ac_Enh;E111,H3K27ac_Enh | E088 | . | yes | . | Arid5a;Cdx2_1;Cdx2_2;Hlx1;Hoxa10;Hoxd10;Mef2_known1 | FGF7 | intronic |
| 15 | rs11635005 | rs17479003 | 0.98 | G | A | 0.16 | E017,6_EnhG;E023,7_Enh;E025,6_EnhG | E025,12_TxEnhW | E017,H3K4me1_Enh;E023,H3K4me1_Enh;E025,H3K4me1_Enh;E026,H3K4me1_Enh;E049,H3K4me1_Enh;E076,H3K4me1_Enh;E078,H3K4me1_Enh;E103,H3K4me1_Enh;E129,H3K4me1_Enh;E023,H3K4me3_Pro;E025,H3K4me3_Pro;E062,H3K4me3_Pro;E023,H3K9ac_Pro;E025,H3K9ac_Pro;E026,H3K27ac_Enh;E049,H3K27ac_Enh;E063,H3K27ac_Enh;E076,H3K27ac_Enh;E129,H3K27ac_Enh |  | . | yes | . | Mef2_known5 | FGF7 | intronic |
| 15 | rs11635005 | rs17400706 | 0.98 | G | A | 0.16 | E017,6_EnhG;E025,3_TxFlnk;E049,6_EnhG;E076,7_Enh;E103,7_Enh;E126,7_Enh;E129,7_Enh | E023,12_TxEnhW;E025,10_TxEnh5;E026,12_TxEnhW;E049,12_TxEnhW;E052,11_TxEnh3 | E008,H3K27ac_Enh;E017,H3K27ac_Enh;E026,H3K27ac_Enh;E049,H3K27ac_Enh;E063,H3K27ac_Enh;E111,H3K27ac_Enh;E129,H3K27ac_Enh;E017,H3K4me1_Enh;E023,H3K4me1_Enh;E025,H3K4me1_Enh;E026,H3K4me1_Enh;E049,H3K4me1_Enh;E063,H3K4me1_Enh;E076,H3K4me1_Enh;E083,H3K4me1_Enh;E088,H3K4me1_Enh;E092,H3K4me1_Enh;E095,H3K4me1_Enh;E103,H3K4me1_Enh;E111,H3K4me1_Enh;E126,H3K4me1_Enh;E128,H3K4me1_Enh;E129,H3K4me1_Enh;E023,H3K4me3_Pro;E025,H3K4me3_Pro;E088,H3K4me3_Pro;E023,H3K9ac_Pro;E025,H3K9ac_Pro |  | . | yes | . | AIRE_1;Gbx2 | FGF7 | intronic |
| 16 | rs4786370 | rs4786370 | 1 | T | C | 0.42 | E001,7_Enh;E005,7_Enh;E007,7_Enh;E015,7_Enh;E016,7_Enh;E019,7_Enh;E020,7_Enh;E022,7_Enh;E034,7_Enh;E037,7_Enh;E039,7_Enh;E040,7_Enh;E041,7_Enh;E042,7_Enh;E043,7_Enh;E044,7_Enh;E045,7_Enh;E046,7_Enh;etc | E001,14_EnhA2;E002,15_EnhAF;E003,15_EnhAF;E004,17_EnhW2;E008,15_EnhAF;E011,15_EnhAF;E012,18_EnhAc;E014,14_EnhA2;E015,14_EnhA2;E016,16_EnhW1;E018,14_EnhA2;E019,15_EnhAF;E020,14_EnhA2;E030,17_EnhW2;E032,17_EnhW2;E033,17_EnhW2;E034,15_EnhAF;E037,14_EnhA2;E038,15_EnhAF;E039,13_EnhA1; etc | E001,H3K4me1_Enh;E003,H3K4me1_Enh;E014,H3K4me1_Enh;E015,H3K4me1_Enh;E016,H3K4me1_Enh;E018,H3K4me1_Enh;E019,H3K4me1_Enh;E020,H3K4me1_Enh;E022,H3K4me1_Enh;E026,H3K4me1_Enh;E027,H3K4me1_Enh;E030,H3K4me1_Enh;E034,H3K4me1_Enh;E037,H3K4me1_Enh;E038,H3K4me1_Enh;E039,H3K4me1_Enh;E040,H3K4me1_Enh;E041,H3K4me1_Enh;E042,H3K4me1_Enh;E043,H3K4me1_Enh;E044,H3K4me1_Enh;E045,H3K4me1_Enh;E046,H3K4me1_Enh;E047,H3K4me1_Enh;E048,H3K4me1_Enh;E056,H3K4me1_Enh;E058,H3K4me1_Enh;E075,H3K4me1_Enh;E077,H3K4me1_Enh;E078,H3K4me1_Enh;E079,H3K4me1_Enh;E084,H3K4me1_Enh;E085,H3K4me1_Enh;E092,H3K4me1_Enh;E093,H3K4me1_Enh;E096,H3K4me1_Enh;E100,H3K4me1_Enh;E101,H3K4me1_Enh;E102,H3K4me1_Enh;E104,H3K4me1_Enh;E105,H3K4me1_Enh;E106,H3K4me1_Enh;E107,H3K4me1_Enh;E108,H3K4me1_Enh;E109,H3K4me1_Enh;E113,H3K4me1_Enh;E114,H3K4me1_Enh; etc | E022;E034;E084;E085 | K562,CEBPB,Stanford,None | yes | yes | HDAC2_disc3 | IL32 | . |
| 16 | rs4786370 | rs55699988 | 1 | G | C | 0.41 | E001,7_Enh;E014,7_Enh;E015,7_Enh;E018,7_Enh;E019,7_Enh;E034,7_Enh;E037,7_Enh;E039,7_Enh;E040,7_Enh;E041,7_Enh;E042,7_Enh;E043,7_Enh;E044,7_Enh;E045,7_Enh;E046,7_Enh;E047,7_Enh;E048,7_Enh;E075,7_Enh;E077,7_Enh;E084,7_Enh;etc | E001,17_EnhW2;E014,17_EnhW2;E015,15_EnhAF;E016,17_EnhW2;E018,17_EnhW2;E019,14_EnhA2;E020,17_EnhW2;E024,19_DNase;E031,18_EnhAc;E032,17_EnhW2;E033,17_EnhW2;E034,15_EnhAF;E037,14_EnhA2;E038,15_EnhAF;E039,15_EnhAF;E040,14_EnhA2;E041,14_EnhA2;E042,15_EnhAF;E043,14_EnhA2;E044,14_EnhA2;E045,15_EnhAF;E046,15_EnhAF;etc | E001,H3K4me1_Enh;E002,H3K4me1_Enh;E003,H3K4me1_Enh;E006,H3K4me1_Enh;E007,H3K4me1_Enh;E014,H3K4me1_Enh;E015,H3K4me1_Enh;E016,H3K4me1_Enh;E018,H3K4me1_Enh;E019,H3K4me1_Enh;E020,H3K4me1_Enh;E022,H3K4me1_Enh;E023,H3K4me1_Enh;E024,H3K4me1_Enh;E026,H3K4me1_Enh;E027,H3K4me1_Enh;E028,H3K4me1_Enh;E034,H3K4me1_Enh;E036,H3K4me1_Enh;E037,H3K4me1_Enh;E038,H3K4me1_Enh;E039,H3K4me1_Enh;E040,H3K4me1_Enh;E041,H3K4me1_Enh;E042,H3K4me1_Enh;E043,H3K4me1_Enh;E044,H3K4me1_Enh;E045,H3K4me1_Enh;E046,H3K4me1_Enh;E047,H3K4me1_Enh;E048,H3K4me1_Enh;E056,H3K4me1_Enh;E058,H3K4me1_Enh;E075,H3K4me1_Enh;E077,H3K4me1_Enh;E078,H3K4me1_Enh;E079,H3K4me1_Enh;E084,H3K4me1_Enh;E085,H3K4me1_Enh;E092,H3K4me1_Enh;E093,H3K4me1_Enh;E096,H3K4me1_Enh;E098,H3K4me1_Enh;E100,H3K4me1_Enh;E101,H3K4me1_Enh;E102,H3K4me1_Enh;E104,H3K4me1_Enh;E106,H3K4me1_Enh;E107,H3K4me1_Enh;E108,H3K4me1_Enh;E109,H3K4me1_Enh;E110,H3K4me1_Enh;etc |  | . | yes | . | LUN-1;Pax-5_known2;SP1_disc3 | IL32 | . |
| 16 | rs4786370 | rs28372698 | 0.99 | T | A | 0.38 | E001,7_Enh;E003,7_Enh;E005,7_Enh;E006,7_Enh;E014,7_Enh;E015,7_Enh;E017,7_Enh;E018,7_Enh;E022,7_Enh;E025,11_BivFlnk;E026,7_Enh;E027,2_TssAFlnk;E032,7_Enh;E033,2_TssAFlnk;E034,11_BivFlnk;E037,2_TssAFlnk;E038,2_TssAFlnk;E039,2_TssAFlnk;E040,2_TssAFlnk;E041,2_TssAFlnk;E042,2_TssAFlnk;E043,2_TssAFlnk;E044,2_TssAFlnk;E045,1_TssA;E046,2_TssAFlnk;E047,2_TssAFlnk;E048,2_TssAFlnk;E049,7_Enh;E052,7_Enh;E055,7_Enh; etc | E001,13_EnhA1;E002,17_EnhW2;E003,16_EnhW1;E004,17_EnhW2;E005,14_EnhA2;E006,2_PromU;E007,16_EnhW1;E008,17_EnhW2;E009,17_EnhW2;E010,17_EnhW2;E011,17_EnhW2;E012,17_EnhW2;E013,17_EnhW2;E014,17_EnhW2;E015,2_PromU;E016,17_EnhW2;E017,2_PromU;E018,16_EnhW1;E019,16_EnhW1;E020,17_EnhW2;E021,17_EnhW2;E022,15_EnhAF;E023,14_EnhA2;E024,17_EnhW2;E025,2_PromU;E026,2_PromU;E027,2_PromU;E028,15_EnhAF;E029,15_EnhAF;E030,15_EnhAF;E031,15_EnhAF;E032,15_EnhAF;E033,3_PromD1;E034,3_PromD1;E035,22_PromP;E036,13_EnhA1;E037,3_PromD1;E038,3_PromD1;E039,3_PromD1;E040,3_PromD1;E041,3_PromD1; etc | E001,H3K4me1_Enh;E002,H3K4me1_Enh;E003,H3K4me1_Enh;E005,H3K4me1_Enh;E006,H3K4me1_Enh;E007,H3K4me1_Enh;E014,H3K4me1_Enh;E015,H3K4me1_Enh;E016,H3K4me1_Enh;E017,H3K4me1_Enh;E018,H3K4me1_Enh;E019,H3K4me1_Enh;E020,H3K4me1_Enh;E021,H3K4me1_Enh;E022,H3K4me1_Enh;E023,H3K4me1_Enh;E024,H3K4me1_Enh;E025,H3K4me1_Enh;E026,H3K4me1_Enh;E027,H3K4me1_Enh;E028,H3K4me1_Enh;E032,H3K4me1_Enh;E033,H3K4me1_Enh;E034,H3K4me1_Enh;E036,H3K4me1_Enh;E037,H3K4me1_Enh;E038,H3K4me1_Enh;E039,H3K4me1_Enh;E040,H3K4me1_Enh;E041,H3K4me1_Enh;E042,H3K4me1_Enh;E043,H3K4me1_Enh;E044,H3K4me1_Enh;E045,H3K4me1_Enh;E046,H3K4me1_Enh;E047,H3K4me1_Enh;E048,H3K4me1_Enh;E049,H3K4me1_Enh;E052,H3K4me1_Enh;E055,H3K4me1_Enh;E056,H3K4me1_Enh;E057,H3K4me1_Enh;E058,H3K4me1_Enh;E062,H3K4me1_Enh;E063,H3K4me1_Enh;E066,H3K4me1_Enh;E075,H3K4me1_Enh;E076,H3K4me1_Enh;E077,H3K4me1_Enh;E078,H3K4me1_Enh;E079,H3K4me1_Enh;E080,H3K4me1_Enh;E084,H3K4me1_Enh;E085,H3K4me1_Enh;E086,H3K4me1_Enh;E089,H3K4me1_Enh;E090,H3K4me1_Enh;E091,H3K4me1_Enh;E092,H3K4me1_Enh;E093,H3K4me1_Enh;E094,H3K4me1_Enh;E095,H3K4me1_Enh;E096,H3K4me1_Enh;E097,H3K4me1_Enh;E098,H3K4me1_Enh;E099,H3K4me1_Enh;E100,H3K4me1_Enh;E101,H3K4me1_Enh;E102,H3K4me1_Enh;E103,H3K4me1_Enh;E104,H3K4me1_Enh;E105,H3K4me1_Enh;E106,H3K4me1_Enh;E107,H3K4me1_Enh;E108,H3K4me1_Enh;E109,H3K4me1_Enh;E110,H3K4me1_Enh;E111,H3K4me1_Enh;E112,H3K4me1_Enh;E113,H3K4me1_Enh;E114,H3K4me1_Enh;E115,H3K4me1_Enh;E116,H3K4me1_Enh;E117,H3K4me1_Enh;E118,H3K4me1_Enh;E119,H3K4me1_Enh;E120,H3K4me1_Enh;E121,H3K4me1_Enh;E122,H3K4me1_Enh;E125,H3K4me1_Enh;E127,H3K4me1_Enh;E129,H3K4me1_Enh;E001,H3K4me3_Pro; etc | E033;E034;E046;E083;E084;E085;E089;E090;E093;E109;E116;E118 | HepG2,HSF1,Stanford,forskolin;HepG2,P300,HudsonAlpha,None;HepG2,PGC1A,Stanford,forskolin | yes | . | Arid5b;BRCA1_known1;RXRA_known5 | IL32 | . |
| 18 | rs1539849 | rs1539849 | 1 | CA | C,CC | NA | E022,7_Enh;E024,7_Enh |  | E001,H3K4me1_Enh;E002,H3K4me1_Enh;E003,H3K4me1_Enh;E008,H3K4me1_Enh;E013,H3K4me1_Enh;E014,H3K4me1_Enh;E015,H3K4me1_Enh;E016,H3K4me1_Enh;E018,H3K4me1_Enh;E019,H3K4me1_Enh;E020,H3K4me1_Enh;E022,H3K4me1_Enh;E024,H3K4me1_Enh;E069,H3K4me1_Enh;E070,H3K4me1_Enh;E080,H3K4me1_Enh;E082,H3K4me1_Enh;E092,H3K4me1_Enh;E003,H3K27ac_Enh;E012,H3K27ac_Enh;E020,H3K27ac_Enh;E067,H3K27ac_Enh;E068,H3K27ac_Enh;E069,H3K27ac_Enh;E072,H3K27ac_Enh;E073,H3K27ac_Enh;E072,H3K9ac_Pro |  | . | . | . | . | CELF4 | intronic |

**Appendix Table 3. Human disease enrichment for *IL32***

| **Disease** | **p value** | **FDR** |
| --- | --- | --- |
| Colitis Ischemic | 6.17E-04 | 0.033669 |
| Mycobacterium avium-intracellulare Infection | 9.87E-04 | 0.033669 |
| Destructive Arthritis | 0.001172478 | 0.033669 |
| Stomach Diseases | 0.001727862 | 0.033669 |
| Cholangitis | 0.00191299 | 0.034996 |
| Active tuberculosis | 0.002962049 | 0.034996 |
| Chronic inflammatory disorder | 0.003023758 | 0.034996 |
| Carcinoma, Large Cell | 0.003887689 | 0.034996 |
| Leishmaniasis | 0.003949398 | 0.034996 |
| Eosinophilic esophagitis | 0.004381364 | 0.034996 |
| Biliary Atresia | 0.005183585 | 0.034996 |
| IMMUNE SUPPRESSION | 0.005738969 | 0.034996 |
| Dermatitis, Allergic Contact | 0.006109226 | 0.034996 |
| Chronic graft-versus-host disease | 0.006541191 | 0.034996 |
| Skin Erosion | 0.006849738 | 0.034996 |
| Giant Cell Arteritis | 0.007343413 | 0.034996 |
| Juvenile rheumatoid arthritis | 0.007837087 | 0.034996 |
| Leprosy | 0.007898797 | 0.034996 |
| Mycosis Fungoides | 0.007960506 | 0.034996 |
| Chronic liver disease | 0.008145634 | 0.034996 |
| Superficial ulcer | 0.008392471 | 0.034996 |
| Sezary Syndrome | 0.008762728 | 0.034996 |
| Progressive cGVHD | 0.010182043 | 0.034996 |
| Progressive Neoplastic Disease | 0.010182043 | 0.034996 |
| Complete atrioventricular block | 0.01042888 | 0.034996 |
| Acute GVH disease | 0.010490589 | 0.034996 |
| Gastritis | 0.010737427 | 0.036074 |
| Malignant neoplasm of gallbladder | 0.011477939 | 0.038522 |
| Gallbladder Carcinoma | 0.013329219 | 0.038522 |
| Hepatitis B Chronic | 0.013452638 | 0.038522 |
| Ankylosing spondylitis | 0.013761185 | 0.038522 |
| Kidney Neoplasm | 0.014008022 | 0.040409 |
| Lymphoma Cutaneous | 0.015242209 | 0.040409 |
| Mesothelioma (malignant (disorder) | 0.015612465 | 0.043909 |
| Juvenile arthritis | 0.017463746 | 0.044587 |
| Hepatitis | 0.018327677 | 0.044587 |
| Helicobacter pylori (H. pylori) infection in conditions classified elsewhere and of unspecified site | 0.019253317 | 0.044587 |
| Helicobacter pylori infection | 0.019253317 | 0.044587 |
| Encephalomyelitis | 0.021413144 | 0.04738 |
| Lung diseases | 0.021536563 | 0.04738 |
